# Supplementary material for: HRCHY-CytoCommunity identifies hierarchical tissue organization in cell-type spatial maps
Source: Nat Commun. 2026 Feb 28;17:3312. doi: 10.1038/s41467-026-70069-z (PMC13065825; doi:10.1038/s41467-026-70069-z)
Supplement: Supplementary file 1 — Supplementary Information [file 41467_2026_70069_MOESM1_ESM.pdf]

HRCHY-CytoCommunity identifies hierarchical tissue organization in cell-type spatial maps

Supplementary Information

Supplementary Notes

Supplementary Note 1. Fine-grained CNs identified in the mouse spleen CODEX and human CRC Visium HD datasets.

We first analyzed the fine-grained CNs identified by HRCHY-CytoCommunity and benchmarked methods in the mouse spleen CODEX dataset. The coarse-grained lymphoid compartment can be subdivided into the marginal zone, B-cell zone and periarteriolar lymphoid sheath (PALS) (Supplementary Fig.2b, right column). We found that HRCHY-CytoCommunity successfully recovered this hierarchical organization. Specifically, among the CNs identified by HRCHY-CytoCommunity, CN-2, CN-3 and CN-4 corresponded to the red pulp; CN-0 and CN-6 corresponded to the marginal zone; CN-1 corresponded to the B-cell zone; and CN-5 corresponded to the PALS (Supplementary Fig.2c, left column). In contrast, the CNs identified by NeST showed poor correspondence with the manually annotated structures (Supplementary Fig.2c, right column). Other non-hierarchical spatial domain detection methods exhibited better alignment with manual annotations than NeST (Supplementary Fig.2d). Among them, the CN boundaries identified by CellCharter were the clearest and most consistent with those detected by HRCHY-CytoCommunity. This consistency can be attributed to CellCharter’s use of scArches, which is a dimension reduction technique that performs well on spatial proteomics data. However, methods originally designed for spatial transcriptomics data, such as GraphST, NicheCompass, and SpaGCN, produced CNs that were intermixed to varying degrees. It was also worth noting that although the fine-grained CNs identified by CellCharter were highly consistent with those by HRCHY-CytoCommunity, CellCharter failed to correctly distinguished the red pulp and lymphoid compartments at the coarse-grained level (Fig. 2c, first column), underscoring the value of explicit hierarchical modeling in tissue structure identification.

We further analyzed the fine-grained CNs identified in the human CRC Visium HD dataset. The results showed that HRCHY-CytoCommunity consistently generated spatially coherent and biologically meaningful CNs across all samples. Specifically, the method subdivided the tumor compartment into a tumor-core CN (CN-6) and several tumor-edge CNs along with immune cell infiltrations (CN-7 and CN-8). Concurrently, the normal tissue compartment was partitioned into CNs enriched with distinct cell types, including an intestinal epithelial-enriched CN (CN-2) and a smooth muscle-enriched CN (CN-0) (Supplementary Fig. 3c, left column). In contrast, the

CNs identified by NeST closely resembled its TCs (Fig. 2f, right column), with the addition of small and fragmented subregions, while a considerable number of spatial bins remained unassigned to any CN (Supplementary Fig. 3c, right column). Among non-hierarchical methods, both NicheCompass and CellCharter detected only a few large CNs and assigned CNs enriched with similar cell types across different samples as distinct CNs, suggesting potential batch effects (Supplementary Fig. 3d).

Taken together, these findings demonstrated that HRCHY-CytoCommunity not only effectively delineates consistent and spatially coherent fined-grained CNs across samples, but more importantly, uncovers biologically meaningful tissue structures that reflect known anatomical and functional organizations.

Supplementary Note 2. Coarse-grained TCs identified in the mouse hypothalamic preoptic region MERFISH and intracerebral hemorrhage Stereo-seq datasets.

We first analyzed the coarse-grained TCs identified in the mouse hypothalamic preoptic region MERFISH dataset. Across all five samples, HRCHY-CytoCommunity consistently identified two TCs per sample, whereas NeST identified only a single TC in each sample (Supplementary Fig. 4c). To assess the biological relevance of these TCs, we calculated cell-type enrichment scores for each compartment (Methods). The results revealed distinct cell-type enrichment patterns across TCs, indicating their functional specialization (Supplementary Fig. 4e). In samples Bregma-0.14, Bregma-0.04, and Bregma+0.06, one TC was predominantly enriched with inhibitory neurons, while the other was enriched with excitatory neurons and non-neuronal cells, including mature oligodendrocytes. Interestingly, in both the Bregma+0.16 and Bregma+0.26 samples, one TC was mainly enriched for mature oligodendrocytes only, while the other was enriched for neurons and other non-neuronal cells. These observations suggest that neuronal and non-neuronal cells can organize into spatially distinct functional structures, and that, depending on the brain region, non-neuronal cells such as mature oligodendrocytes, exhibit differential interactions with excitatory and inhibitory neurons. This finding is consistent with established knowledge that non-neuronal cells play a role in modulating neuronal excitability in both physiological and pathophysiological brain processes [1–3].

In the mouse intracerebral hemorrhage (ICH) Stereo-seq dataset, HRCHY-CytoCommunity consistently identified three biologically meaningful TCs across most samples: TC-0 (brain stem), TC-1 (cortex), and TC-2 (striatum) (Supplementary Fig. 6c, left column). Among the other benchmarked methods, only NicheCompass recovered a similar structural organization, whereas the remaining methods failed to robustly identify these compartments across multiple samples (Supplementary Figs. 6c and d).

Taken together, these results demonstrate that HRCHY-CytoCommunity effectively reconstructs biologically relevant coarse-grained TCs from both imaging-based and sequencing-based spatial transcriptomics data, capturing consistent functional structures across diverse brain regions and experimental conditions.

Supplementary Note 3. Ablation studies of HRCHY-CytoCommunity.

To systematically evaluate the contribution of each key component of the HRCHY-CytoCommunity model, we performed ablation studies on both simulated and real spatial omics datasets. The evaluated components included: (1) the consistency and balance regularization terms, (2) the adaptive edge-pruning strategy, and (3) the adaptive α-scheduling strategy. For real multi-sample datasets, we selected the sample with the largest number of cells to conduct the ablation experiments.

On the simulated data (Supplementary Note 7), for coarse-grained TC identification, the best performance was achieved only when both consistency and balance regularization were applied together, confirming their effectiveness in preventing cluster collapse and improving model stability (Supplementary Fig. 13a, left two columns). Similarly, the adaptive edge-pruning strategy outperformed both extreme configurations (i.e., preserving all edges and removing all edges), demonstrating its utility in filtering trivial connections among fine-grained CNs (Supplementary Fig. 13b, left two columns). However, the adaptive α-scheduling strategy showed little effect on the simulated data (Supplementary Fig. 13c). For fine-grained CN identification, none of the three components provided substantial improvement, and the performance of HRCHY-CytoCommunity maintained stable and excellent. This is consistent with expectations, as these components were primarily designed for preventing suboptimal coarse-grained TC identification.

On real spatial omics data, consistent with simulations, models incorporating consistency and balance regularization, or adaptive edge pruning, yielded the best performance in coarse-grained TC identification using the mouse spleen CODEX and the human CRC Visium HD datasets (Supplementary Figs. 14a and b). Notably, different from the simulated data, the adaptive α-scheduling strategy improved performance across both real datasets (Supplementary Fig. 14c), suggesting that it facilitates progressive hierarchical optimization under complex biological structures. For fine-grained CN identification using the mouse hypothalamic preoptic region MERFISH and the mouse ICH Stereo-seq datasets, consistent with simulations, none of the components provided notable gains, while performance remained stable across all configurations (Supplementary Fig. 15).

In summary, these ablation studies demonstrate that the three evaluated components of HRCHY-CytoCommunity play critical and complementary roles in coarse-grained TC identification. In contrast, for fine-grained CN identification, the model maintains robust performance across varying component settings.

Supplementary Note 4. Sensitivity analysis of hyperparameters in HRCHY-CytoCommunity.

To assess the robustness of HRCHY-CytoCommunity to hyperparameter settings, we performed sensitivity analysis on both simulated and real spatial omics datasets. The tested hyperparameters included: (1) the DropNode rate (fraction of nodes whose features are masked during consistency regularization training), (2) λ_consis (weight

of the consistency regularization term), (3) λ_balance (weight of the balance regularization term), (4) the number of neighbors *K* in the KNN graph (cell-cell proximity graph) construction, and (5) the number of perturbations used for generating perturbed cell-cell proximity graphs.

On the simulated data (Supplementary Note 7), HRCHY-CytoCommunity exhibited stable performance across a broad range of hyperparameter values (Supplementary Fig. 16). A slight performance decrease was observed at high DropNode rates, suggesting that excessive feature masking may hinder the model's ability to accurately capture graph topology, thereby affecting hierarchical tissue structure assignments. For all other hyperparameters, both AMI and Macro-F1 scores for coarse-grained TC and fine-grained CN identification remained consistently high, highlighting the model's strong robustness.

Similar trends were observed on real spatial omics data. For coarse-grained TC identification using the mouse spleen CODEX and the human CRC Visium HD datasets, performance remained largely stable across hyperparameter variations, with moderate sensitivity to λ_consis and λ_balance (Supplementary Fig. 17). Excessively high DropNode rates led to performance decrease, likely due to the loss of critical node feature information. The adaptive edge-pruning strategy ensured robustness to different *K* values. Increasing the number of graph perturbations did not substantially alter the results. For fine-grained CN identification using the mouse hypothalamic preoptic region MERFISH and the mouse ICH Stereo-seq datasets, performance remained nearly unchanged across all tested hyperparameter settings (Supplementary Fig. 18), further confirming the model's stability.

In summary, these results demonstrate that HRCHY-CytoCommunity achieves robust performance without requiring extensive hyperparameter tuning. Although hyperparameters such as regularization weights exhibit some influence on coarse-grained TC identification, the framework maintains stable accuracy across both simulated and real datasets, supporting its practical applicability to diverse spatial omics platforms.

Supplementary Note 5. Robustness assessment of HRCHY-CytoCommunity.

To systematically evaluate the robustness of HRCHY-CytoCommunity under varying conditions of cell-type annotation quality, we conducted experiments on two spatial omics datasets with complex cell-type compositions, assessing performance under two scenarios: (1) increasing rates of cell-type label inaccuracies, and (2) different levels of annotation resolution.

We first evaluated robustness to cell-type label inaccuracies using the mouse hypothalamic preoptic region MERFISH dataset. By randomly perturbing 10% to 50% of the cell-type annotations, we found that the identified hierarchical tissue structures remained highly consistent until the label error rate exceeded 40% (Supplementary Fig. 19a). We further quantified the accuracy of identified fine-grained CNs using AMI and Macro-F1 scores. As expected, performance showed a

moderate decline as the label error rate increased, but remained well-preserved under reasonable noise levels (Supplementary Figs. 19b and c), indicating the model's ability to tolerate cell-type annotation errors.

We next assessed robustness to cell-type annotation resolution by comparing results obtained using 15 versus 9 cell-type annotations (Supplementary Figs. 19d and e). HRCHY-CytoCommunity consistently recovered stable hierarchical tissue structures, with an average robustness score of 0.673 across samples (Supplementary Fig. 19f), demonstrating insensitivity to changes in cell-type annotation granularity.

Similar evaluations were performed on the human TNBC MIBI-TOF dataset. Hierarchical tissue structures remained highly consistent across cell-type label perturbation rates (Supplementary Fig. 20a). We also measured the accuracy of coarse-grained TC identification by calculating the proportion of neoplastic and immune cells correctly assigned to their corresponding TCs under varying noise levels. Performance was largely preserved under reasonable levels of annotation noise, with only a moderate decrease as the label error rate increased (Supplementary Fig. 20b). When comparing results obtained from 17 versus 11 cell-type annotations, fine-grained CNs showed slight variations, but coarse-grained TCs remained highly consistent (Supplementary Figs. 20c and d). Quantitatively, the model achieved an average robustness score of 0.590 across samples under different cell-type annotation resolutions (Supplementary Fig. 20e).

In summary, these results demonstrate that HRCHY-CytoCommunity is strongly robust to both label inaccuracies and variations in annotation resolution, supporting its reliability for applications where cell-type annotations may be incomplete or noisy.

Supplementary Note 6. Scalability and speed analysis.

We evaluated the runtime and memory efficiency of HRCHY-CytoCommunity using the largest Visium HD sample (P2CRC; 473,318 spots, 18,085 genes). To assess scalability, we generated four down-sampled subsets containing 1k, 10k, 100k, and 473k spots, respectively. The benchmarking was conducted on a server equipped with an Intel Xeon Gold-6154 3.00 GHz CPU (72 cores), 512 GB RAM, and an NVIDIA RTX3090 GPU (24 GB memory).

HRCHY-CytoCommunity was evaluated in two configurations: (i) the base model (without consistency and balance regularization), and (ii) the full model (with both regularization terms). We also compared its performance against six other spatial domain detection methods, including NeST, CellCharter, NicheCompass, GraphST, SpaGCN, and SpaSEG. Notably, NeST failed to identify valid gene hotspots on the 1k-spot subset. GraphST, which uses dense adjacency representations, exceeded available memory (>500 GB) at 100k spots. Similarly, SpaGCN exceeded 500 GB memory on the full 473k-spot dataset.

In terms of runtime, both models of HRCHY-CytoCommunity achieved outstanding performance across all dataset sizes (Supplementary Fig. 21a). In particular, the base model achieved the fastest training speed, completing hierarchical tissue structure

identification on the full 473k-spot dataset in only 1.39 minutes. The full model, which involves repeated stochastic perturbations (DropNode), completed the same dataset in 8.02 minutes, ranking third after the base model and SpaSEG. Among other methods, NeST and SpaSEG also showed strong scalability, with SpaSEG exhibiting nearly constant runtime (3.94 minutes on the full 473k-spot dataset) regardless of data size.

To provide a more detailed comparison, we decomposed runtime into training and clustering stages (Supplementary Figs. 21b-e). Methods were categorized into one-step and two-step models. One-step models included HRCHY-CytoCommunity, SpaGCN, and SpaSEG, which directly output cluster assignments during training. Two-step models included GraphST, NicheCompass, and CellCharter, which first learn embeddings of cells or spots and then apply clustering. NeST, being non-neural-network-based, was considered as clustering-only. Results indicated that the additional clustering step in two-step models introduced computational cost, potentially becoming a bottleneck on large-scale datasets (Supplementary Fig. 21e). However, non-hierarchical one-step models (e.g., SpaGCN and SpaSEG) require training separate models for different hierarchical resolutions, introducing additional cost for multi-scale analysis.

In terms of memory usage, both models of HRCHY-CytoCommunity exhibited nearly linear memory growth with data size increasing (Supplementary Fig. 22a), reflecting great scalability. This efficiency stems from our implementation that relies exclusively on sparse matrix operations, resulting in a space complexity of $O(E)$, where E denotes the number of graph edges. In contrast, most other methods (except SpaSEG and CellCharter) showed steep memory growth with data scale increasing.

We further decomposed the total memory consumption into RAM usage and GPU memory usage (Supplementary Figs. 22b-e). Interestingly, although NicheCompass required relatively high total memory, it employed mini-batch training to maintain a relatively low GPU memory usage (6.21 GB on the full 473k-spot dataset). This characteristic allowed NicheCompass to remain trainable on GPUs, even for large-scale datasets.

In summary, these results demonstrate that HRCHY-CytoCommunity achieves highly competitive scalability in both runtime and memory efficiency, supporting its practical application to increasingly large and high-resolution spatial omics datasets.

Supplementary Note 7. Experimental details for reproducibility.

Hardware environment:

All experiment were performed on a server equipped with an Intel Xeon Gold-6154 3.00 GHz CPU (72 cores), 512 GB RAM, and an NVIDIA RTX3090 GPU (24 GB memory).

Datasets:

Mouse spleen CODEX dataset. This dataset includes three samples. For coarse-grained TC identification, we manually merged the periarteriolar lymphoid sheath (PALS), B-cell zone, and marginal zone into the lymphoid compartment. The resulting annotations served as ground truth for evaluating coarse-grained TCs. Cell type annotations were used as input to HRCHY-CytoCommunity. Giotto Suite was excluded from this dataset analysis due to excessive memory requirement (> 500 GB). SpaSEG was also excluded because it lacks a preprocessing module for spatial proteomics data.

Human CRC Visium HD dataset. This dataset includes three samples. Quality control involved filtering out 8 μm bins with UMI counts below 50 and mitochondrial gene expression ratio exceeding 30%. Cell type annotation of each 8 μm bin (UnsupervisedL1) were download from https://github.com/10XGenomics/HumanColonCancer_VisiumHD and used as input to HRCHY-CytoCommunity. To evaluate coarse-grained TCs, the 50 μm periphery and non-tumor tissue regions were merged into a single normal tissue compartment, which was treated as ground truth. SpaGCN, GraphST, and Giotto Suite were excluded due to memory requirement > 500 GB.

Mouse hypothalamic preoptic region MERFISH dataset. This dataset includes five samples. We used 17-cell-type annotations as input to HRCHY-CytoCommunity.

Mouse ICH Stereo-seq dataset. We analyzed samples Naive-1, H3-1, H6-1, H12-1, D1-3, D3-3, D7-4, D14-4, and D28-4 from the original study. The annotation CT_II was used as input to HRCHY-CytoCommunity. Giotto Suite and SpaSEG were excluded due to memory requirement > 500 GB.

Mouse hippocampus Slide-seq V2 dataset. We analyzed the Puck_200115_08 sample in this study. Cell type deconvolution was performed using RCTD.

Human breast cancer Visium V1 dataset. This dataset includes one slice with 3,987 spots. Cell type deconvolution was performed using RCTD.

Human TNBC MIBI-TOF dataset. This spatial proteomics dataset includes 41 TNBC tumor samples. Among these, 15 samples exhibited compartmentalized neoplastic-immune architectures, with immune cells spatially separated from neoplastic cells.

Human breast cancer IMC dataset. This dataset contains 281 patients with long-term survival data. 18 patients were excluded from the survival analysis due to no hierarchical tissue structure identified.

Simulated data. We adapted the simulation code from NicheCompass (https://github.com/Lotfollahi-lab/nichecompass-reproducibility/blob/main/analysis/data_simulation/data_simulation.R) to generate synthetic spatial transcriptomics data. We simulated 10,000 cells distributed across two TCs and eight CNs with varying cell-type compositions. The TC design was inspired by the human TNBC MIBI-TOF dataset. Specifically, one TC was immune cell (cell type 1)-dominated and the other was neoplastic cell (cell type 8)-dominated.

Each TC contained multiple CNs with distinct cell-type compositional patterns, yielding a ground truth hierarchical structure of two TCs and eight CNs. (Supplementary Fig. 23) The cell-type composition profiles for CNs were provided in Supplementary Table 3.

Supplementary Note 8. Extension of HRCHY-CytoCommunity to three or more hierarchical levels.

The core HRCHY-CytoCommunity framework described in the main text operates on a two-level hierarchy for clarity. However, the model is inherently designed to be extended to an arbitrary number of hierarchical levels, denoted as *K*. This note outlines the generalized formulation for identifying *K*-level hierarchical tissue structures.

The model constructs the hierarchy iteratively from the bottom to up (i.e., level 1 to level *K*). For each level *k*=1,2, ..., *K*−1, the following steps are performed:

(1) Node representation learning

A graph neural network (GNN) is used to learn a *d*-dimensional node representation matrix $Z^{(k)}$ at level *k*:

$$Z^{(k)} = \mathrm{ReLU}(\mathrm{GNN_k}\left(X^{(k)}, A^{(k)}; \theta_{\mathrm{GNN}_k}\right)) \tag{1}$$

where each row in $Z^{(k)}$ is a learned *d*-dimensional representation vector for each node (e.g., a cell or a pooled node from a previous level). At level *k*, the graph contains c_k nodes. $X^{(k)} \in \mathbb{R}^{c_k \times d}$ represents the node feature matrix. $A^{(k)}$ is the adjacency matrix of the graph. θ_{GNN_k} represents trainable parameters of the GNN.

(2) Soft hierarchical structure assignment

A fully-connected layer followed by a Softmax function produces the soft assignment matrix $S^{(k)}$, which specifies the probabilistic membership of nodes at level *k* to the c_{k+1} pooled nodes (clusters) at level *k*+1.

$$S^{(k)} = \mathrm{Softmax}\left(\mathrm{FC}_k\left(Z^{(k)}; \theta_{\mathrm{FC}_k}\right)\right) \tag{2}$$

where each element of $S^{(k)} \in \mathbb{R}^{C_k \times C_{k+1}}$ represents the probability of a node at level *k* belonging to a cluster at level *k+1*.

(3) Differentiable graph pooling and edge pruning

The graph is coarsened to form the input for level *k*+1. The feature matrix $X^{(k+1)}$ and pooled adjacency matrix A^{pool} are computed as:

$$X^{(k+1)} = {S^{(k)}}^T Z^{(k)} \tag{3}$$

$$A^{(k+1)} = {S^{(k)}}^T A^{(k)} S^{(k)} \quad (4)$$

Self-loops are removed and the adjacency matrix is normalized as:

$$\hat{A} = A^{(k+1)} - I_{C_k} diag(A^{(k+1)}) \quad (5)$$

$$A^{pool} = \widehat{D}^{-\frac{1}{2}} \hat{A} \widehat{D}^{-\frac{1}{2}} \quad (6)$$

where $\widehat{D}$ is the degree matrix of $\hat{A}$. To prevent over-smoothing, edges with weights below an adaptive threshold $T_{edge\_pruning} = \frac{1}{C_k - 1}$ are pruned.

(4) Loss function for the base module

The base model is trained end-to-end using a unified loss function that simultaneously optimizes the cluster assignments at all *K* levels. The loss at level *k* is defined as:

$$L_k = -\frac{\sum_{j=1}^{c_k} ({S^{(k)}}^T A^{(k)} S^{(k)})_{jj}}{\sum_{j=1}^{c_k} ({S^{(k)}}^T D^{(k)} S^{(k)})_{jj}} + \left\| \frac{{S^{(k)}}^T S^{(k)}}{\left\| {S^{(k)}}^T S^{(k)} \right\|_F} - \frac{I_{C_k}}{\sqrt{c_k}} \right\|_F \quad (7)$$

where $D^{(k)}$ is the degree matrix of $A^{(k)}$. The total loss is a weighted sum of the losses across all *K* levels:

$$L_{Base} = \sum_{k=1}^{K} \alpha_k \times L_k \quad (8)$$

where α_k is a weight parameter balancing the contribution of the loss from each hierarchical level.

This generalized *K*-level formulation demonstrates the extensibility of HRCHY-CytoCommunity, providing a principled and scalable framework for uncovering multi-scale spatial organization in tissues in any depths.

Supplementary Note 9. Identification of ambiguous structures in hierarchical tissue organization.

While HRCHY-CytoCommunity enforces discrete (hard) hierarchical assignments to ensure interpretability and consistency in downstream analyses. biological tissues often contain overlapping or transitional regions, where cells may exhibit features of multiple TCs or CNs. To enable users to identify and analyze such ambiguous structures, we designed an entropy-based uncertainty measure that quantifies the confidence of cell-to-structure (e.g., CN) assignments.

$$uncertainty\_score_i = \frac{-\sum_{k=0}^{c_1 - 1} s_{i,k}^{(1)} \log s_{i,k}^{(1)}}{\log c_1} \quad (9)$$

where $uncertainty\_score_i$ denotes the normalized entropy of soft assignment distribution of cell *i*, $s_{i,k}^{(1)}$ is the probability of cell *i* belonging to CN-*k*, and c_1 is the

pre-defined number of CNs. This score ranges from 0 to 1, with values near 0 indicating high confidence in assignment and values near 1 reflecting high ambiguity.

We applied this measure to the mouse hippocampus Slide-seq V2 dataset. Cells with higher uncertainty scores were predominantly located at the boundaries between identified CNs (Supplementary Fig. 24), suggesting that these regions may represent transitional zones or areas of overlapping cellular identities.

This functionality provides users with a systematic approach to evaluate the ambiguity of cell-to-structure assignments. Cells with high uncertainty scores may indicate potential multi-membership or transitional zones within the hierarchical tissue structures, offering deeper insights into spatial tissue organization beyond discrete partitioning.

Supplementary Figures

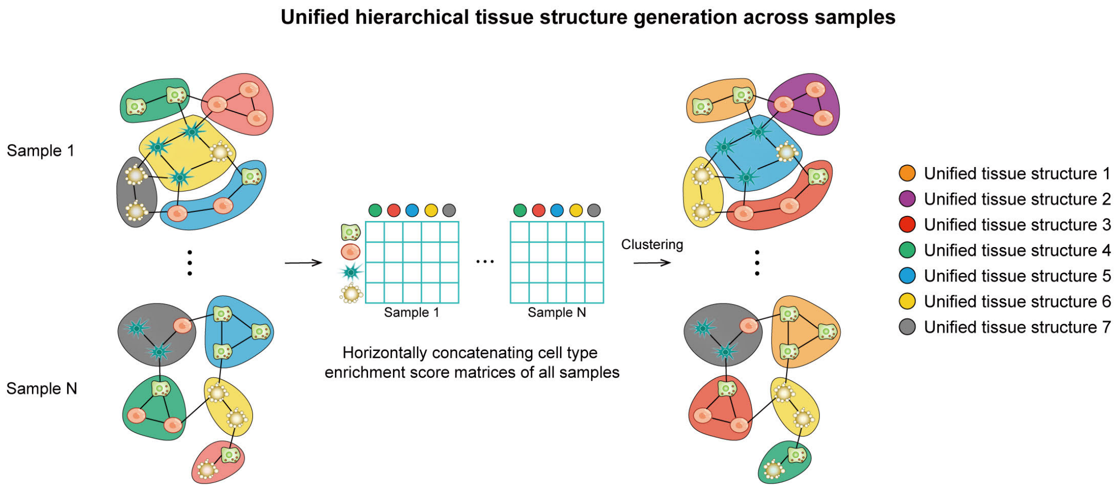

Supplementary Figure 1. Optional clustering-based module for cross-sample integration analysis. An optional clustering-based module designed to generate a unified set of hierarchical tissue structures for cross-sample integration analysis. For each sample, the cell-type enrichment score matrix of identified tissue structures is first computed. Then these matrices are then horizontally concatenated to construct a composite feature matrix. A clustering algorithm is subsequently applied to this composite matrix to group tissue structures from different samples into unified hierarchical tissue structures. This process enables the generation of a consistent and comparable hierarchical tissue structure set across samples, facilitating downstream cross-sample comparative analyses.

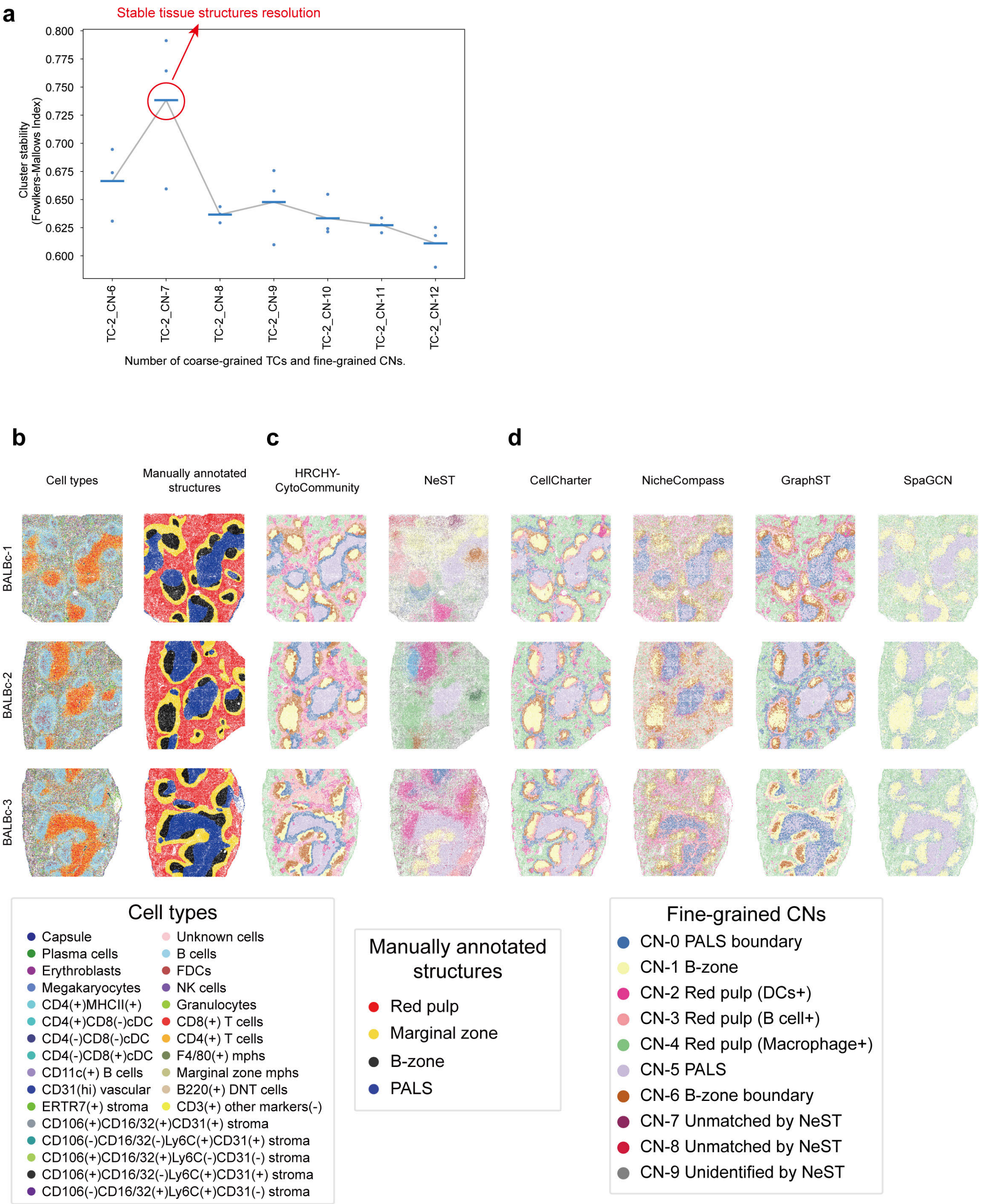

Supplementary Figure 2. Spatial maps of fine-grained CNs in the mouse spleen CODEX dataset. (a) Cluster stability measure used by HRCHY-CytoCommunity to determine the optimal numbers of coarse-grained TCs and fine-grained CNs. Each point represents the Fowlkes-Mallows Index-based cluster stability for an individual sample. Horizontal bars indicate the mean across n = 3 samples. Mean values under each parameter setting are connected by grey solid lines. The red circle marks the selected stable resolution of tissue structures. **(b)** Spatial maps showing the distribution of cell types (left) and manually annotated structures (right). PALS, periarteriolar lymphoid sheath. **(c)** Fine-grained CNs identified by HRCHY-CytoCommunity (left) and NeST (right). **(d)** Fine-grained CNs identified by non-hierarchical tissue structure identification methods, including CellCharter,

NicheCompass, GraphST, and SpaGCN. Unidentified indicates cells not assigned to any CN. Unmatched denotes CNs that could not be aligned with manual annotations. Source data are provided as a Source Data file.

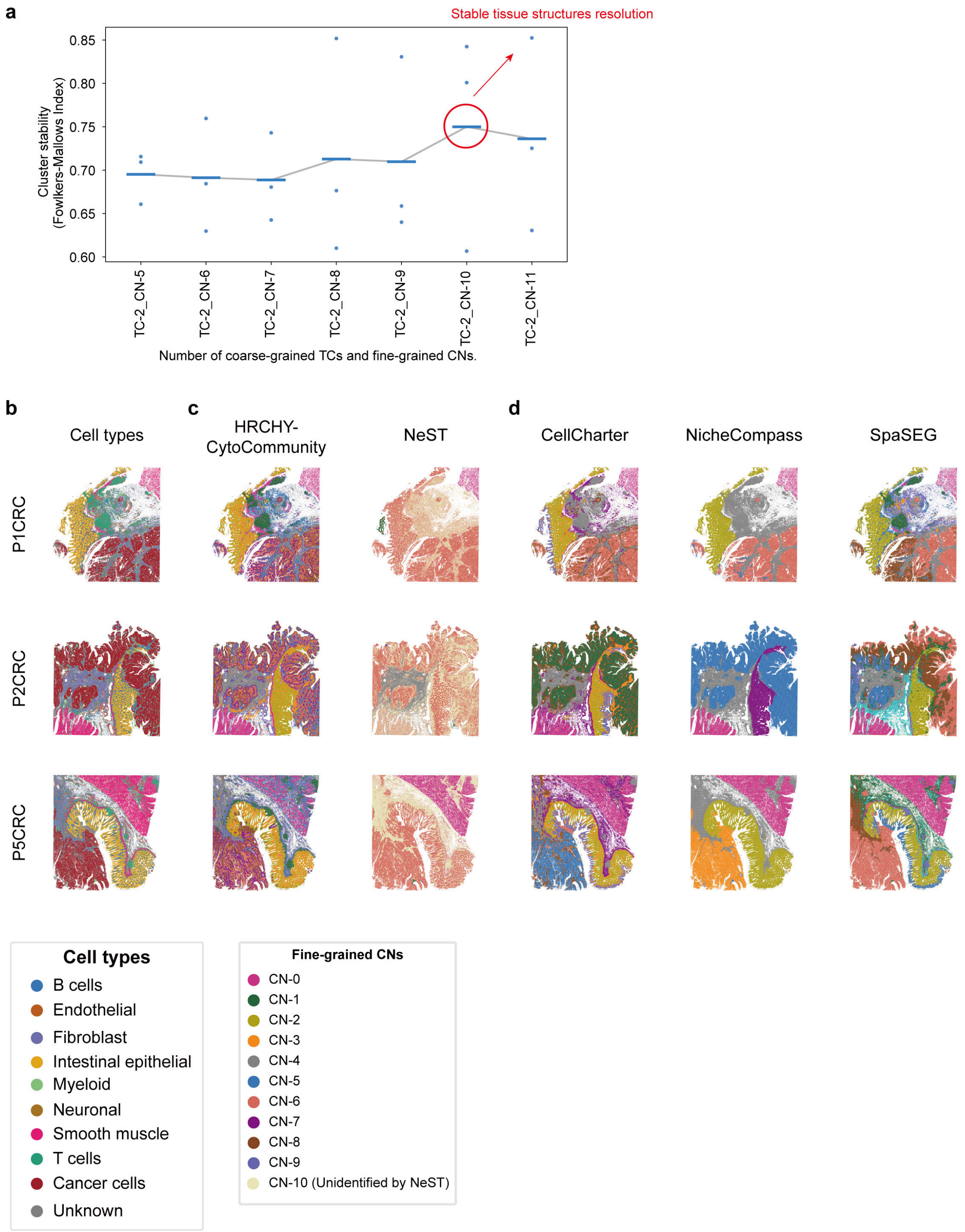

Supplementary Figure 3. Spatial maps of fine-grained CNs in the human CRC Visium HD dataset. (a) Cluster stability measure used by HRCHY-CytoCommunity to determine the optimal numbers of coarse-grained TCs and fine-grained CNs. Each point represents the Fowlkes-Mallows Index-based cluster stability for an individual sample. Horizontal bars indicate the mean across n = 3 samples. Mean values under each parameter setting are connected by grey solid lines. The red circle marks the selected stable resolution of tissue structures. **(b)** Spatial maps (8 µm bins) showing the distribution of cell types. **(c)** Fine-grained CNs identified by HRCHY-CytoCommunity (left) and NeST (right). **(d)** Fine-grained CNs identified by non-

hierarchical tissue structure identification methods, including CellCharter, NicheCompass, and SpaSEG. Unidentified indicates cells not assigned to any CN. Source data are provided as a Source Data file.

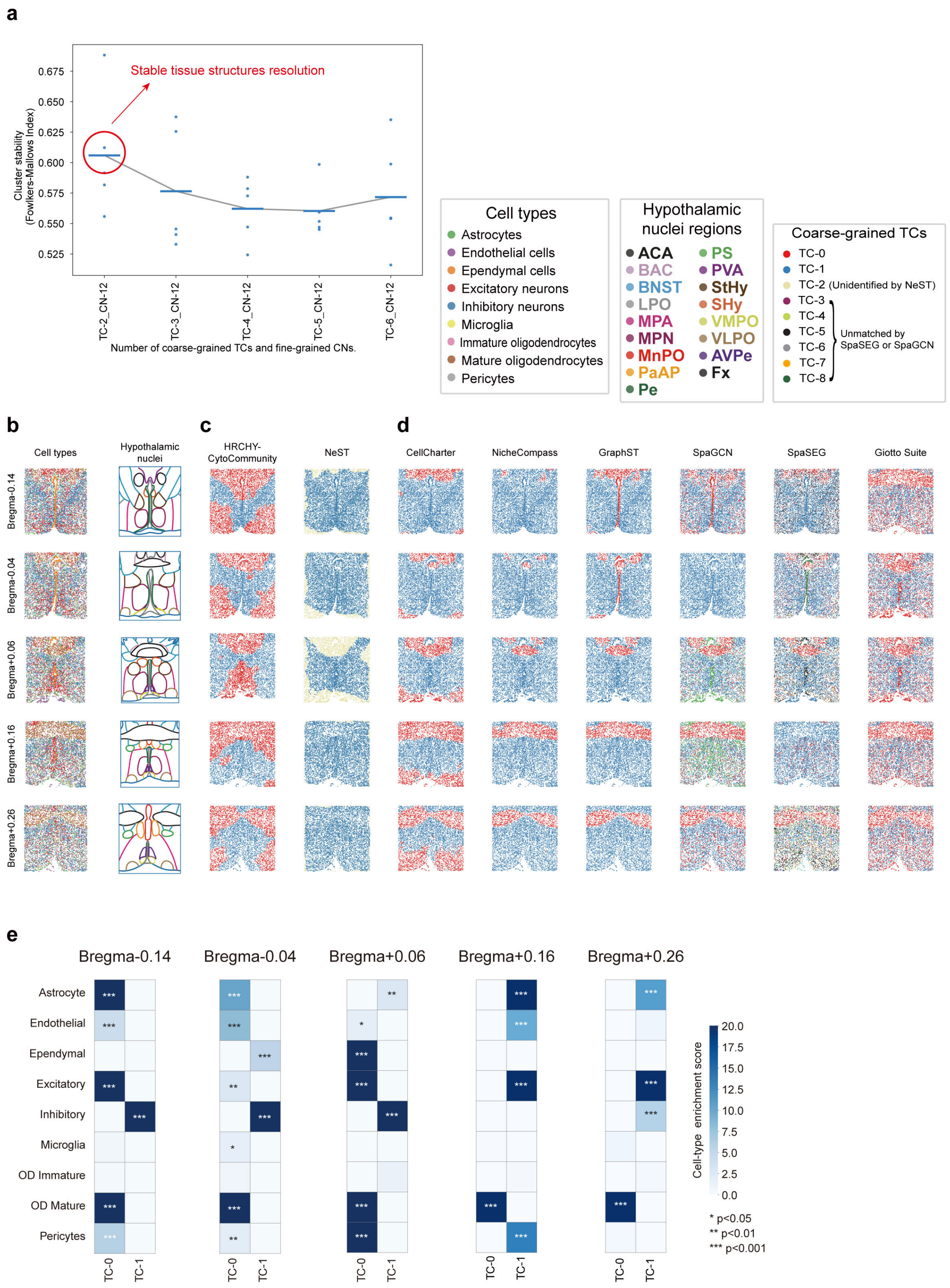

Supplementary Figure 4. Spatial maps of coarse-grained TCs in the mouse hypothalamic preoptic region MERFISH dataset. (a) Cluster stability measure used by HRCHY-CytoCommunity to determine the optimal numbers of coarse-grained TCs and fine-grained CNs. Each point represents the Fowlkes-Mallows Index-based cluster stability for an individual sample. Horizontal bars indicate the mean across n = 5 samples. Mean values under each parameter setting are connected by grey solid lines. The red circle marks the selected stable resolution of tissue

structures. **(b)** Spatial maps showing the distribution of cell types (left) and manually annotated structures (right). **(c)** Coarse-grained TCs identified by HRCHY-CytoCommunity (left) and NeST (right). **(d)** Coarse-grained TCs identified by non-hierarchical tissue structure identification methods, including CellCharter, NicheCompass, GraphST, SpaGCN, SpaSEG and Giotto Suite. Unidentified indicates cells not assigned to any TC. Unmatched denotes TCs that could not be aligned with manual annotations. **(e)** Heatmaps showing the cell-type enrichment scores for each coarse-grained TC in all samples. Cell-type enrichment score was defined as $-\log_{10}(P\text{-value})$. *P*-values were computed using hypergeometric tests and adjusted with the Benjamini-Hochberg method. OD Immature, Immature oligodendrocyte; OD Mature, Mature oligodendrocyte. Source data are provided as a Source Data file.

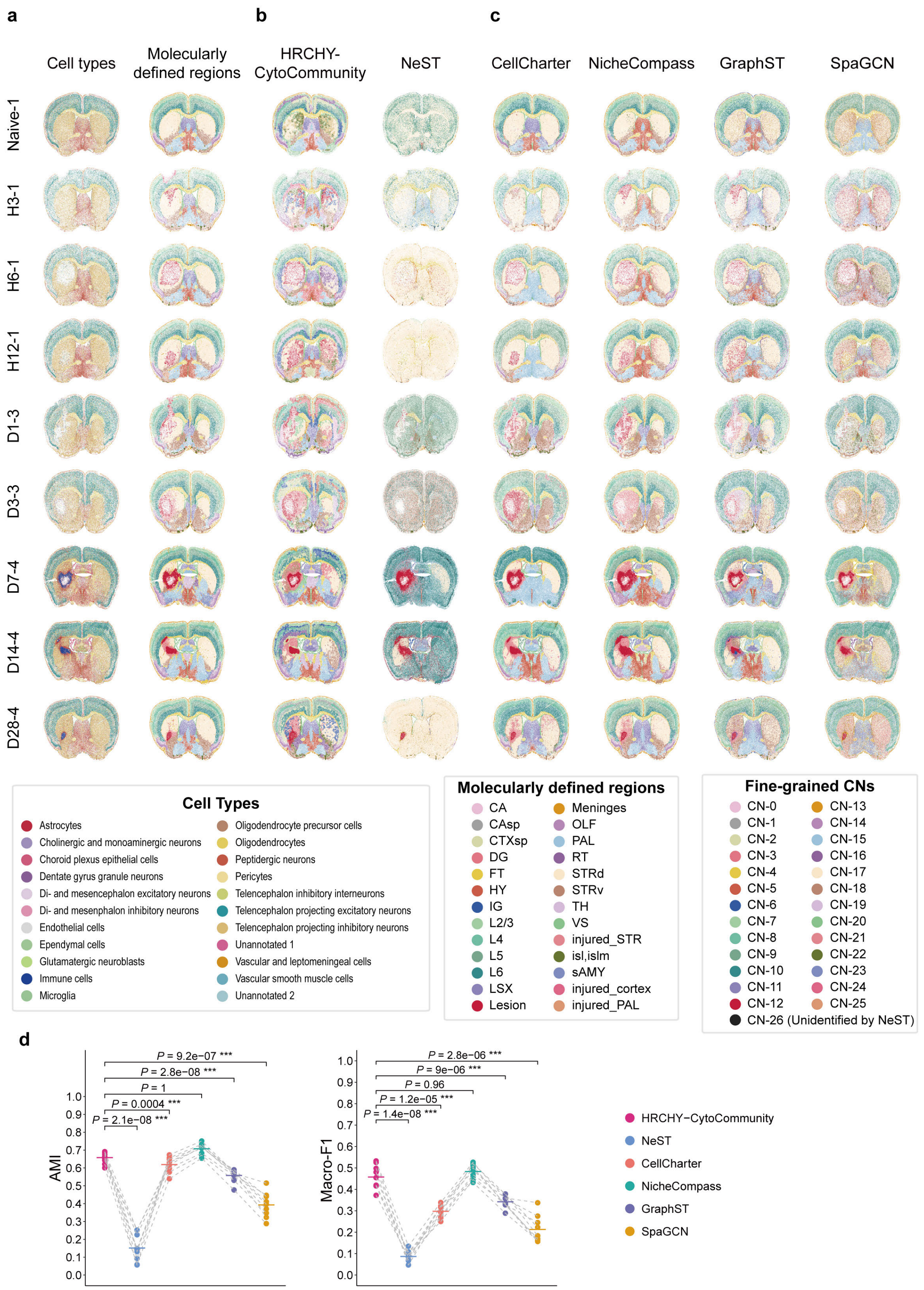

Supplementary Figure 5. Performance evaluation of HRCHY-CytoCommunity using the mouse ICH Stereo-seq dataset. (a) Spatial maps showing the distribution of cell types (left) and molecularly defined regions (right). **(b)** Fine-grained CNs identified by HRCHY-CytoCommunity (left) and NeST (right). **(c)** Fine-grained CNs identified by non-hierarchical tissue structure identification methods, including CellCharter, NicheCompass, GraphST, and SpaGCN. Unidentified indicates cells not

assigned to any CN. **(d)** AMI and Macro-F1 scores calculated using molecularly defined regions. Each point corresponds to the performance on an individual sample, with horizontal bars indicating the mean performance across n = 9 samples. Points from the same samples are connected by grey dashed lines. *P*-values were calculated using a one-sided paired *t*-test. *, *P*-value < 0.05; **, *P*-value < 0.01; ***, *P*-value < 0.001. L2/3, cortical layer 2 and cortical layer 3; L4, cortical layer 4; L5, cortical layer 5; L6, cortical layer 6; Lesion, Lesion region; injured_STR, injured striatum region; injured_cortex, injured cortex region; injured_PAL, injured pallidum region; DG, dentate gyrus; FT, fiber tracts; HY, hypothalamus; OLF, olfactory areas; MH, medial habenula; CA, Ammon's horn; STRd, striatum dorsal region; STRv, striatum ventral region; TH, thalamus; VS, ventricular systems; isl, islm, islands of Calleja and major island of Calleja; PAL, pallidum; RT, reticular nucleus of the thalamus; CAsp, field Ammon's horn, pyramidal layer; LSX, lateral septal complex; sAMY, striatum-like amygdala nuclei; CTXsp, cortical subplate. Source data are provided as a Source Data file.

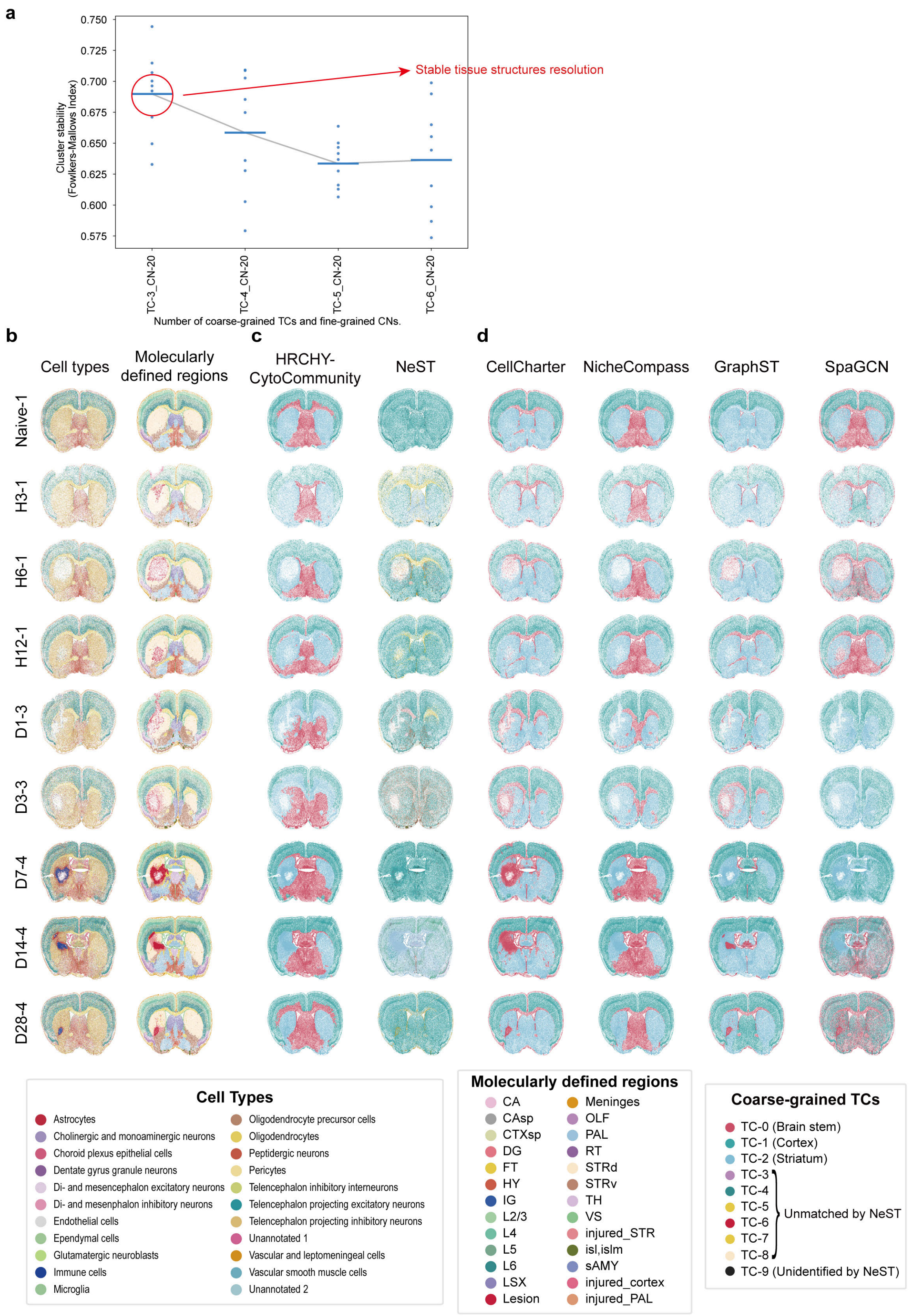

Supplementary Figure 6. Spatial maps of coarse-grained TCs in the mouse ICH Stereo-seq dataset. (a) Cluster stability measure used by HRCHY-CytoCommunity to determine the optimal numbers of coarse-grained TCs and fine-grained CNs. Each point represents the Fowlkes-Mallows Index-based cluster stability for an individual sample. Horizontal bars indicate the mean across n = 9 samples. Mean values under

each parameter setting are connected by grey solid lines. The red circle marks the selected stable resolution of tissue structures. **(b)** Spatial maps showing the distribution of cell types (left) and molecularly defined regions (right), **(c)** Coarse-grained TCs identified by HRCHY-CytoCommunity (left) and NeST (right). **(d)** Coarse-grained TCs identified by non-hierarchical tissue structure identification methods, including CellCharter, NicheCompass, GraphST, and SpaGCN. Unidentified indicates cells not assigned to any TC. Unmatched denotes CNs that could not be aligned with manual annotations.L2/3, cortical layer 2 and cortical layer 3; L4, cortical layer 4; L5, cortical layer 5; L6, cortical layer 6; Lesion, Lesion region; injured_STR, injured striatum region; injured_cortex, injured cortex region; injured_PAL, injured pallidum region; DG, dentate gyrus; FT, fiber tracts; HY, hypothalamus; OLF, olfactory areas; MH, medial habenula; CA, Ammon's horn; STRd, striatum dorsal region; STRv, striatum ventral region; TH, thalamus; VS, ventricular systems; isl, islm, islands of Calleja and major island of Calleja; PAL, pallidum; RT, reticular nucleus of the thalamus; CAsp, field Ammon's horn, pyramidal layer; LSX, lateral septal complex; sAMY, striatum-like amygdala nuclei; CTXsp, cortical subplate. Source data are provided as a Source Data file.

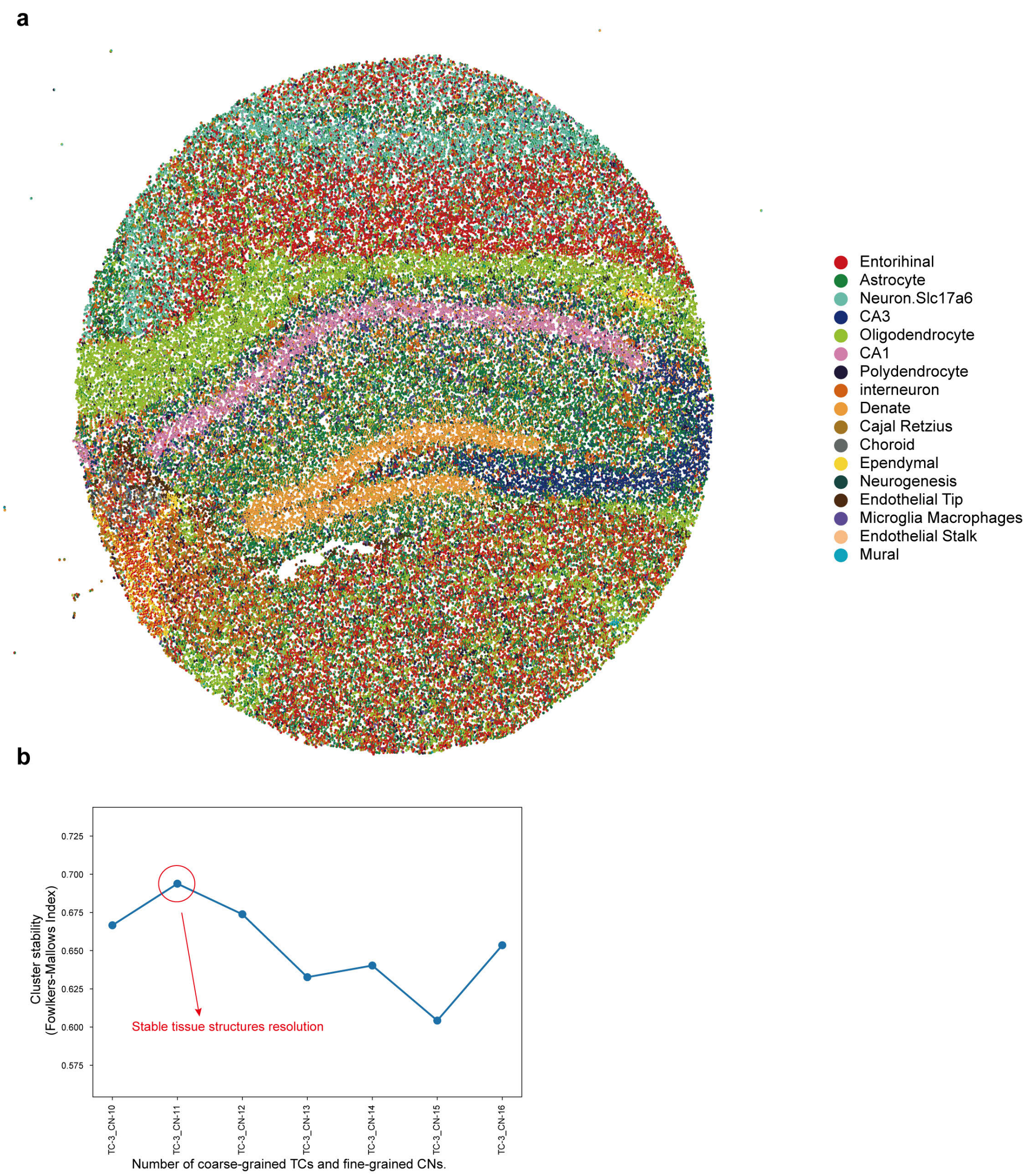

Supplementary Figure 7. Cell-type map and cluster stability across different parameter settings on the mouse hippocampus Slide-seq V2 dataset. (a) Spatial map of pie charts showing cell-type compositions inferred by RCTD. **(b)** Cluster stability measure used by HRCHY-CytoCommunity to determine the optimal numbers of coarse-grained TCs and fine-grained CNs. The Fowlkes-Mallows Index-based cluster stability values under each parameter setting are connected by solid lines. The red circle marks the selected stable resolution of tissue structures. Source data are provided as a Source Data file.

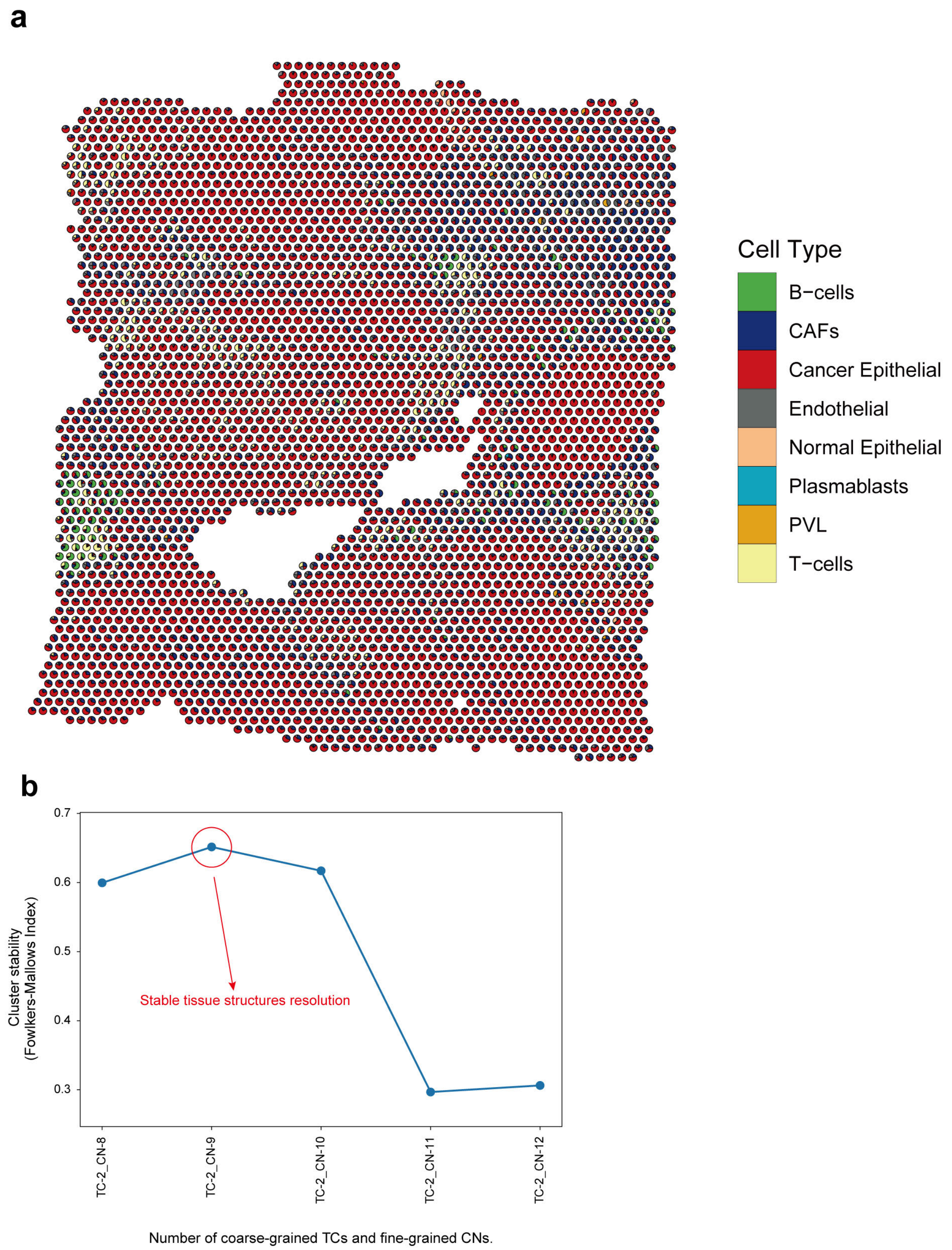

Supplementary Figure 8. Cell-type map and cluster stability across different parameter settings on the human breast cancer Visium V1 dataset. (a) Spatial map of pie charts showing cell-type compositions inferred by RCTD. **(b)** Cluster stability measure used by HRCHY-CytoCommunity to determine the optimal numbers of coarse-grained TCs and fine-grained CNs. The Fowlkes-Mallows Index-based cluster stability values under each parameter setting are connected by solid lines. The red circle marks the selected stable resolution of tissue structures. Source data are provided as a Source Data file.

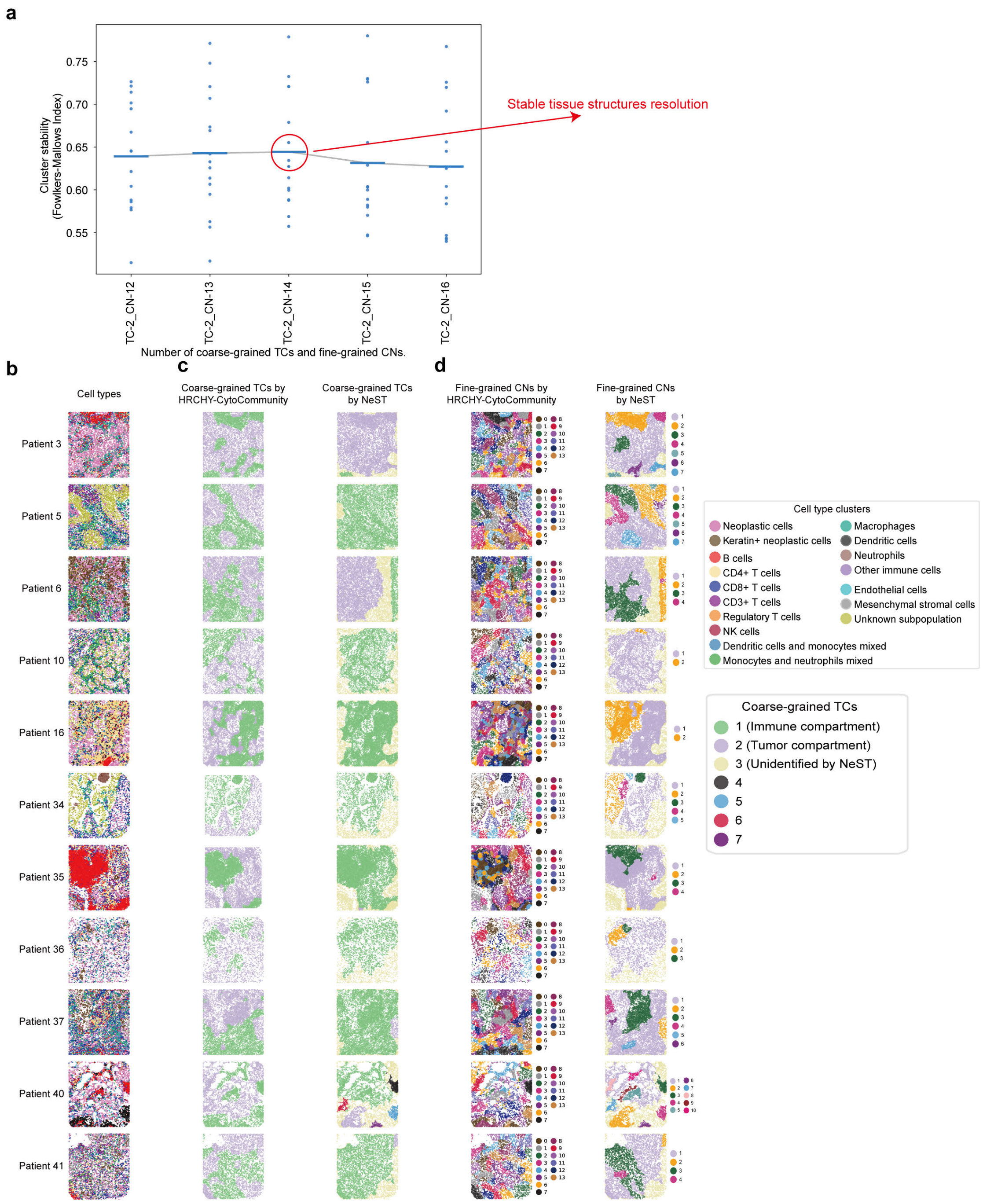

Supplementary Figure 9. Spatial maps of coarse-grained TCs and fine-grained CNs in the triple-negative breast cancer MIBI-TOF dataset (except patients 4, 9, 28 and 32). (a) Cluster stability measure used by HRCHY-CytoCommunity to determine the optimal numbers of coarse-grained TCs and fine-grained CNs. Each point represents the Fowlkes-Mallows Index-based cluster stability for an individual sample. Horizontal bars indicate the mean across n = 15 samples. Mean values under each parameter setting are connected by grey solid lines. The red circle marks the selected stable resolution of tissue structures. **(b)** Single-cell spatial maps showing the distribution of cell types. **(c)** Coarse-grained TCs identified by HRCHY-CytoCommunity (left column) and NeST (right column). **(d)** Fine-grained CNs identified by HRCHY-CytoCommunity (left column) and NeST (right column).

Unidentified indicates cells not assigned to any TC or CN. Source data are provided as a Source Data file.

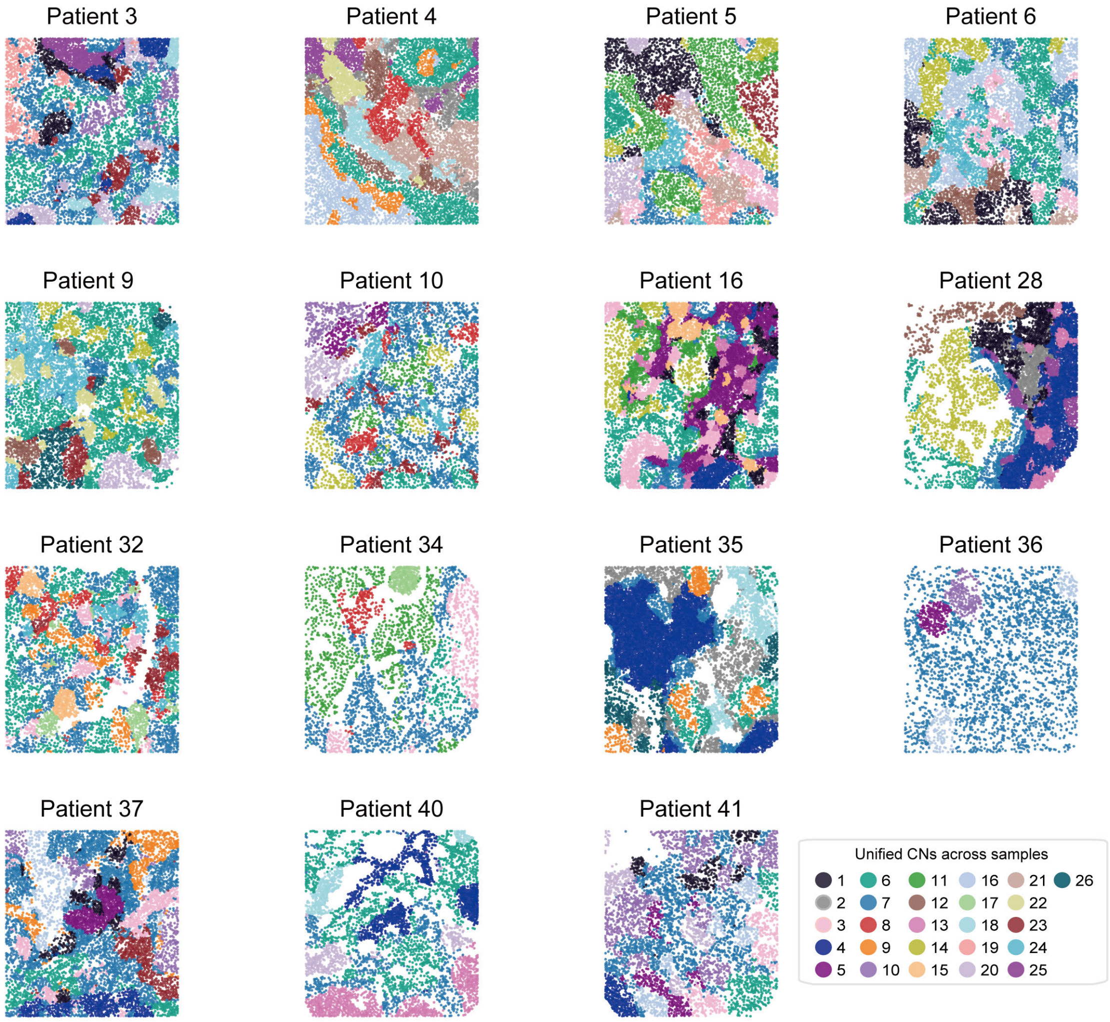

Supplementary Figure 10. Spatial maps of unified CNs identified by HRCHY-CytoCommunity in the TNBC MIBI-TOF dataset.

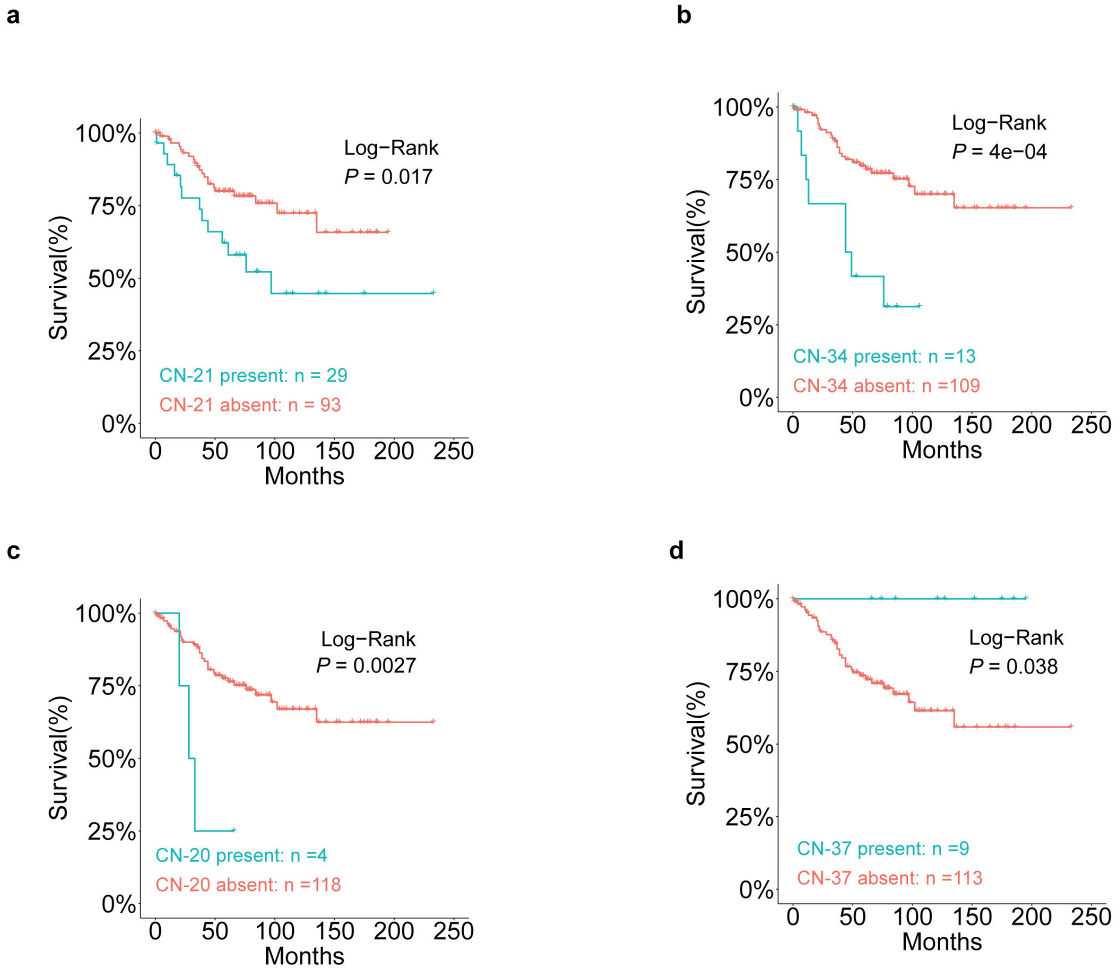

Supplementary Figure 11. Complementary results of survival analysis for the breast cancer IMC dataset. (a-d) Kaplan-Meier survival curves of patients within Group 1, categorized into two groups based on **(a)** unified CN-21, **(b)** unified CN-34, **(c)** unified CN-20, and **(d)** unified CN-37, respectively. *P*-values were calculated using the log-rank tests.

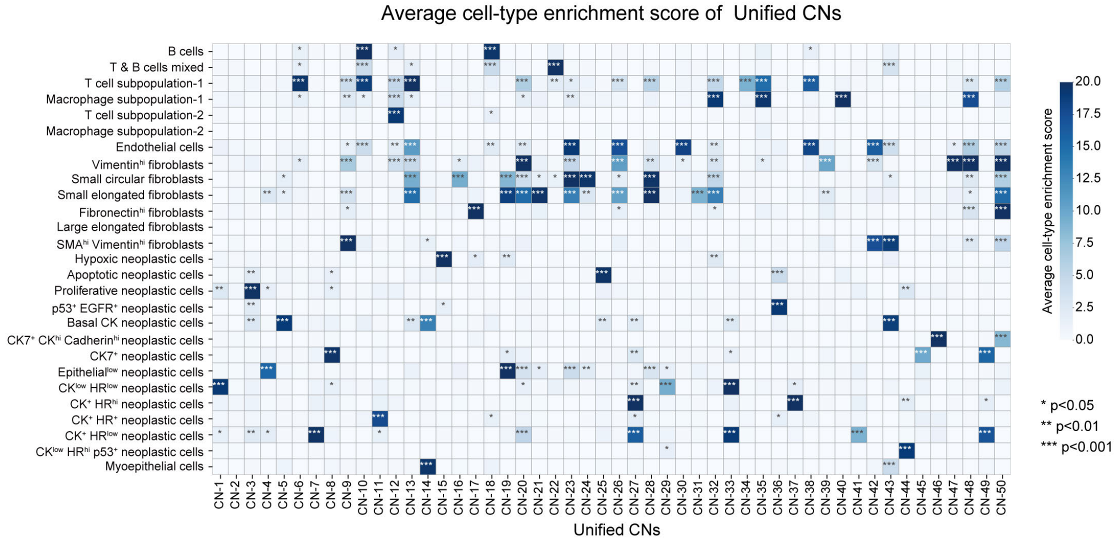

Supplementary Figure 12. Average cell-type enrichment scores of unified CNs in the breast cancer IMC dataset. The heatmap showing the average cell-type enrichment scores for each unified CN. Cell-type enrichment score was defined as $-\log_{10}$(*P*-value). *P*-values were computed using hypergeometric tests and adjusted with the Benjamini-Hochberg method. *, *P*-value < 0.05; **, *P*-value < 0.01; ***, *P*-value < 0.001. Source data are provided as a Source Data file.

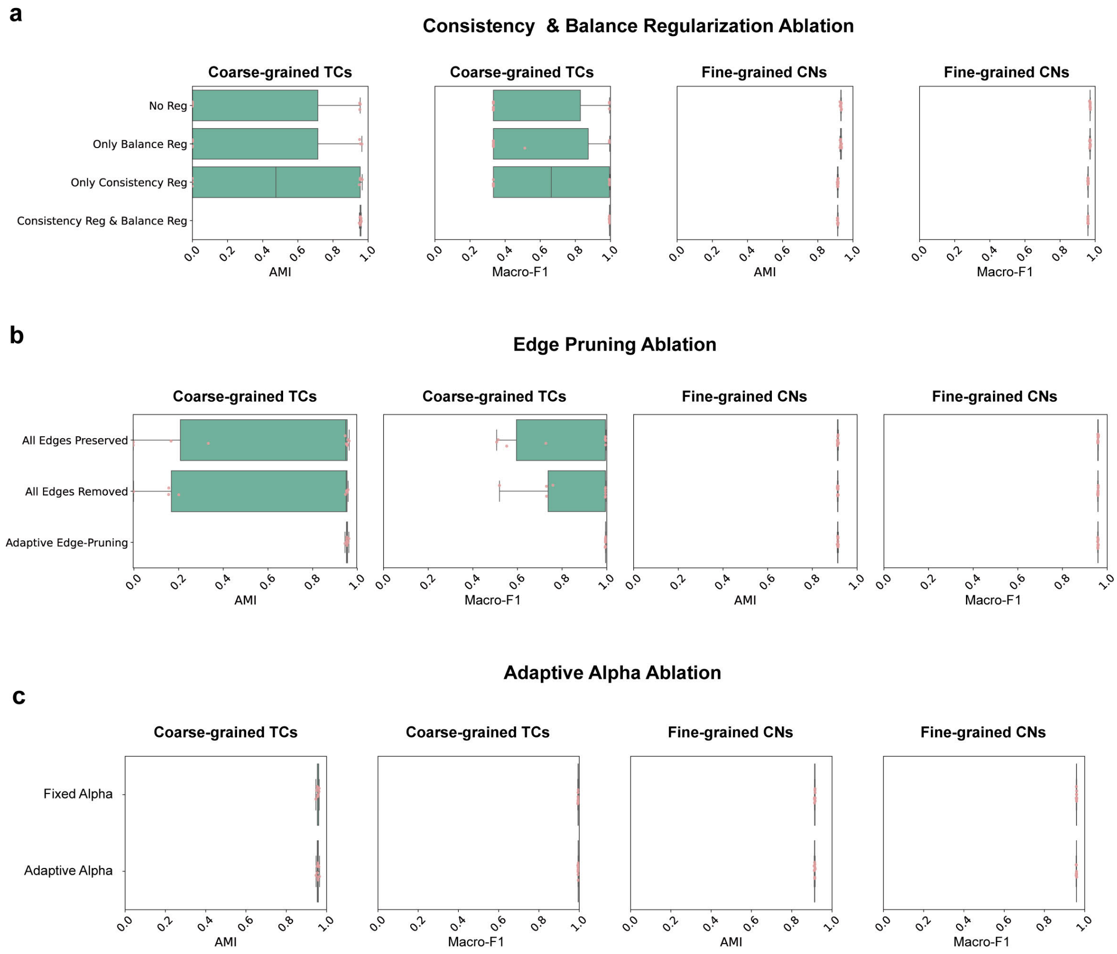

Supplementary Figure 13. Ablation study of key components in HRCHY-CytoCommunity using the simulated dataset. Hierarchical tissue structure identification under different model configurations. Each configuration was run 10 times with different random seeds. Boxplot elements are defined as: center line, median; box limits, upper and lower quartiles; whiskers, 1.5× interquartile range. Performances are quantified by Adjusted Mutual Information (AMI) and Macro-F1 scores. Each point corresponds to the performance of one run. Ablation studies were performed on **(a)** consistency and balance regularization terms, **(b)** the adaptive edge-pruning strategy, and **(c)** the adaptive α-scheduling strategy. Source data are provided as a Source Data file.

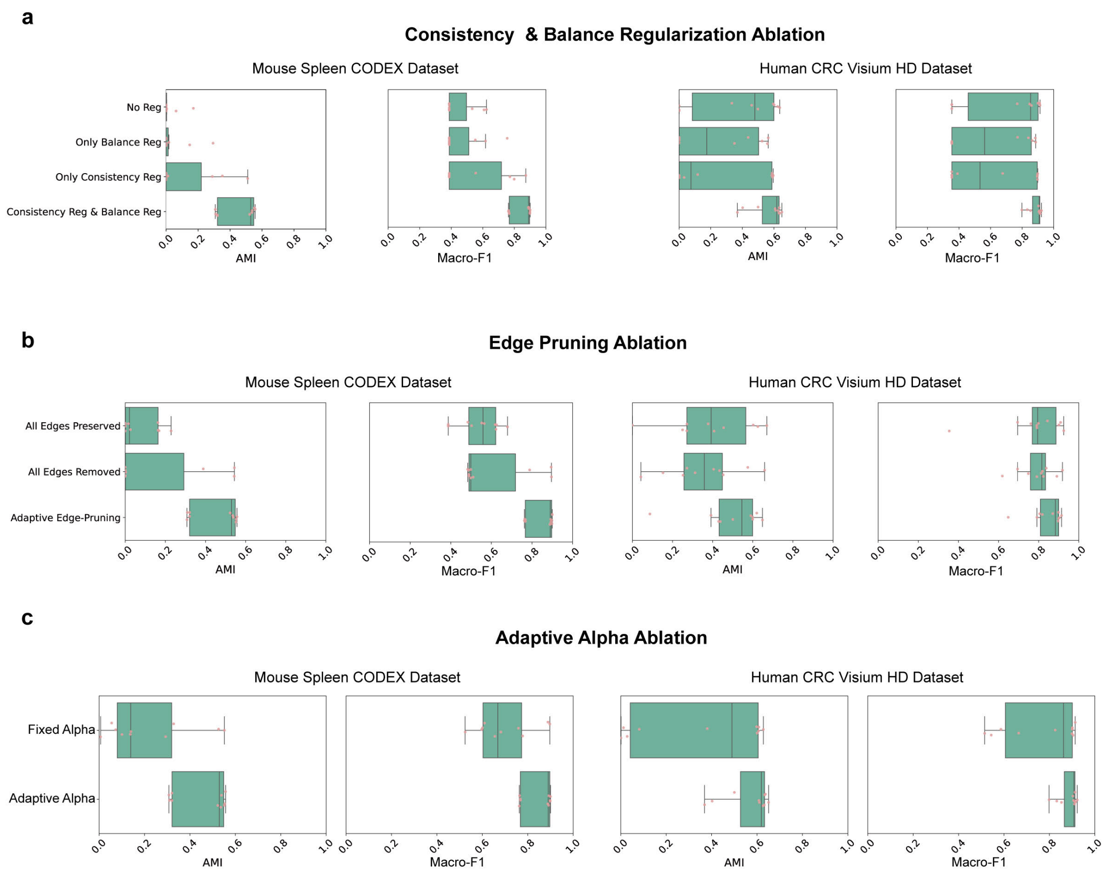

Supplementary Figure 14. Ablation study of key components in HRCHY-CytoCommunity on coarse-grained TC identification using real spatial omics datasets. Hierarchical tissue structure identification under different model configurations using the mouse spleen CODEX dataset (imaging-based) and the human colorectal cancer (CRC) Visium HD dataset (sequencing-based). Each run was run 10 times with different random seeds. Boxplot elements are defined as: center line, median; box limits, upper and lower quartiles; whiskers, 1.5× interquartile range. Performances are quantified by Adjusted Mutual Information (AMI) and Macro-F1 scores. Each point corresponds to the performance of one run. Ablation studies were performed on **(a)** consistency and balance regularization terms, **(b)** the adaptive edge-pruning strategy, and **(c)** the adaptive α-scheduling strategy. Source data are provided as a Source Data file.

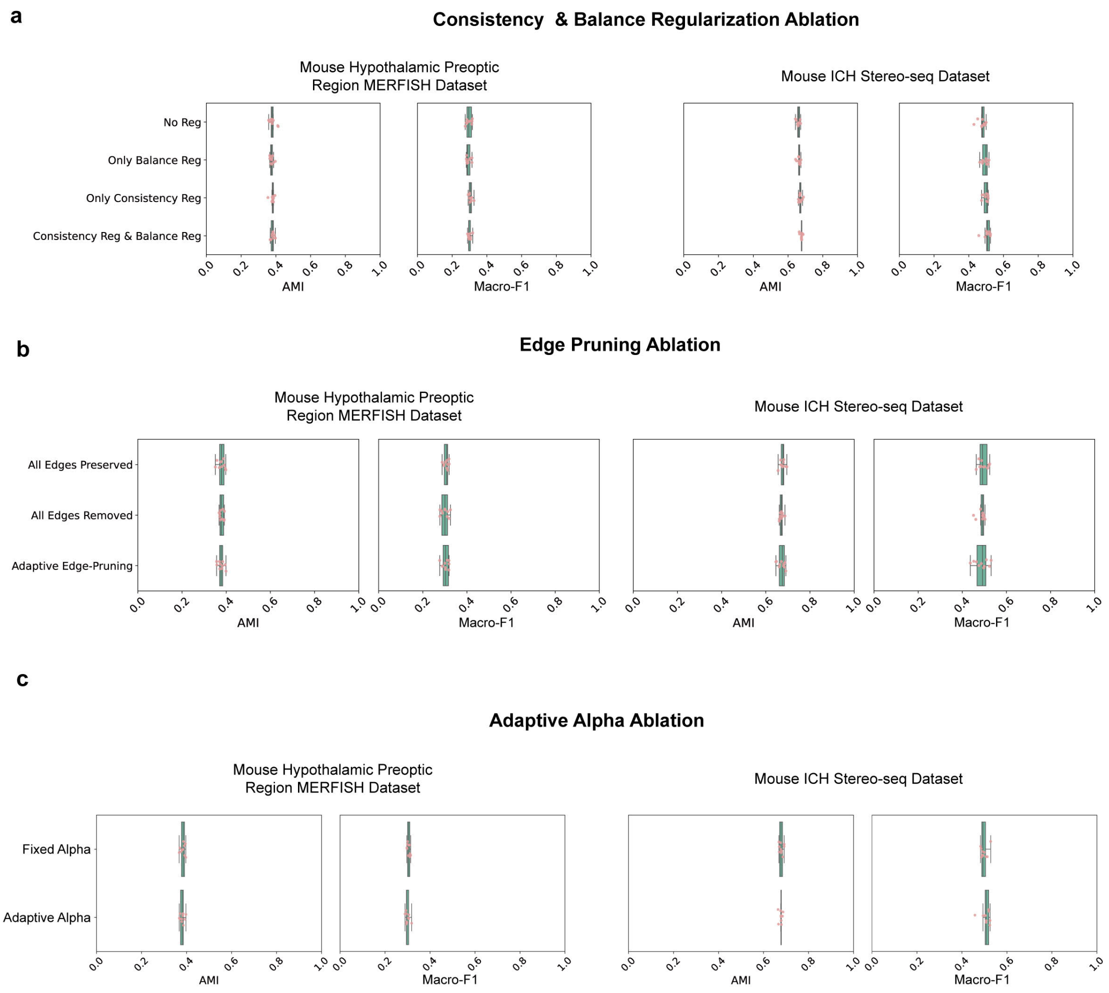

Supplementary Figure 15. Ablation study of key components in HRCHY-CytoCommunity on fine-grained CN identification using real spatial omics datasets. Hierarchical tissue structure identification under different model configurations using the mouse hypothalamic preoptic region MERFISH dataset (imaging-based) and the mouse intracerebral hemorrhage (ICH) Stereo-seq dataset (sequencing-based). Each configuration was run 10 times with different random seeds. Boxplot elements are defined as: center line, median; box limits, upper and lower quartiles; whiskers, 1.5× interquartile range. Performances are quantified by Adjusted Mutual Information (AMI) and Macro-F1 scores. Each point corresponds to the performance of one run. Ablation studies were performed on **(a)** consistency and balance regularization terms, **(b)** the adaptive edge-pruning strategy, and **(c)** the adaptive α-scheduling strategy. Source data are provided as a Source Data file.

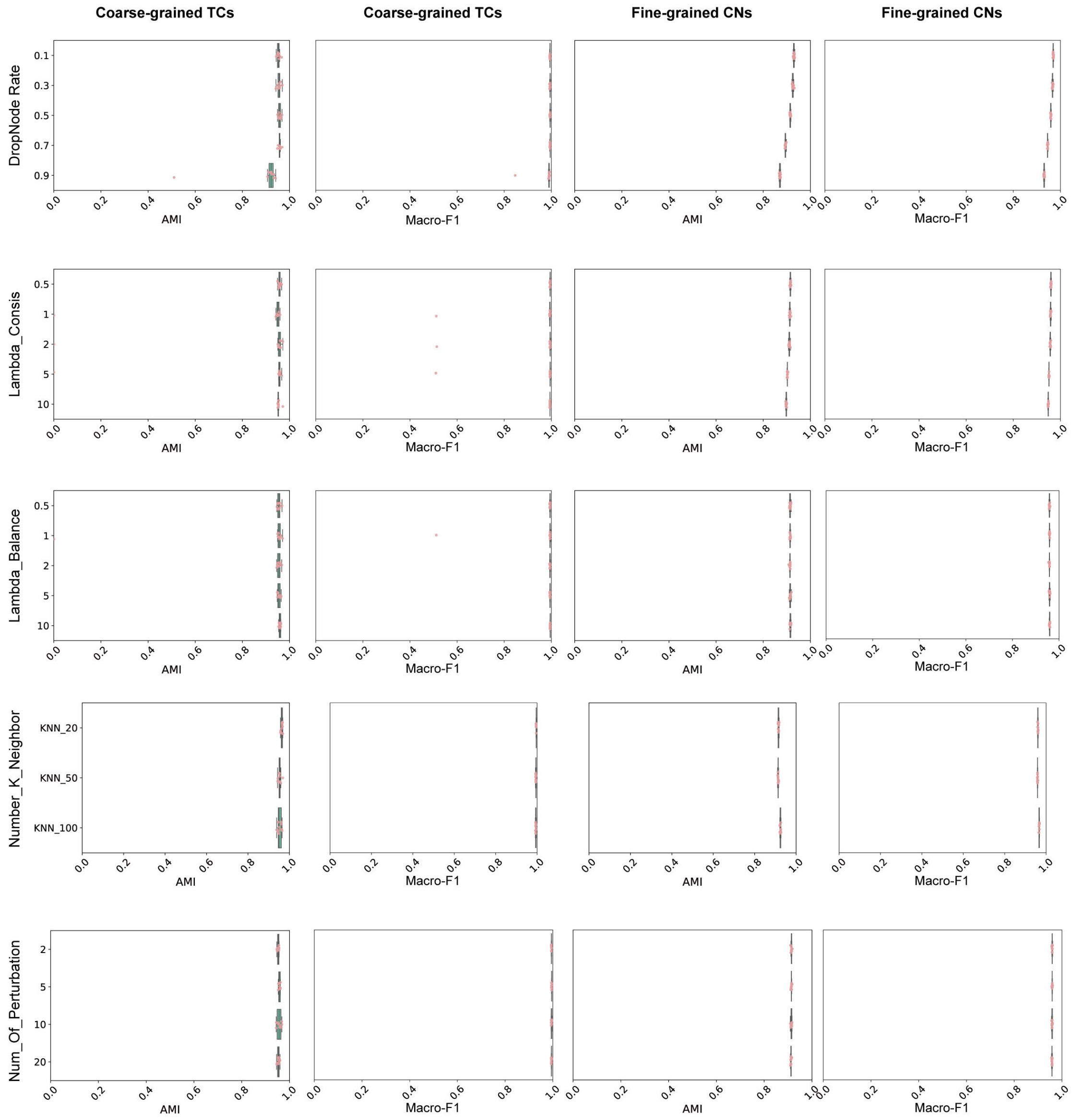

Supplementary Figure 16. Sensitivity analysis of hyperparameters in HRCHY-CytoCommunity using the simulated dataset. Hierarchical tissue structure identification under different model configurations. Each configuration was run 10 times with different random seeds. Boxplot elements are defined as: center line, median; box limits, upper and lower quartiles; whiskers, 1.5× interquartile range. Performances are quantified by Adjusted Mutual Information (AMI) and Macro-F1 scores. Each point corresponds to the performance of one run. This sensitivity analysis includes five hyperparameters: (1) the DropNode rate (fraction of nodes whose features are masked during consistency regularization training), (2) λ_consis (weight of the consistency regularization term), (3) λ_balance (weight of the balance regularization term), (4) the number of neighbors *K* in the KNN graph (cell-cell proximity graph) construction, and (5) the number of perturbations used for generating perturbed cell-cell proximity graphs. Source data are provided as a Source Data file.

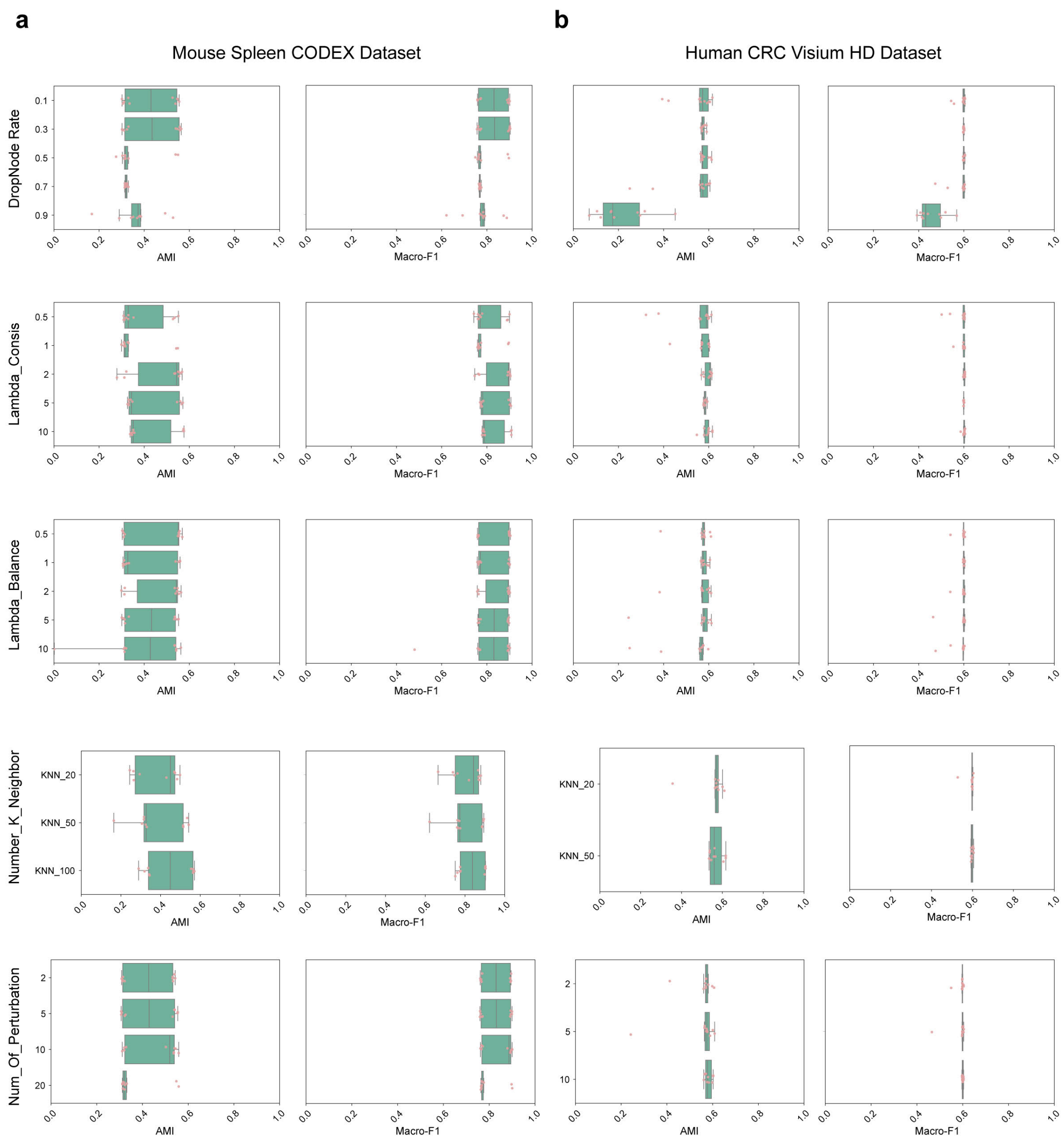

Supplementary Figure 17. Sensitivity analysis of hyperparameters in HRCHY-CytoCommunity on coarse-grained TC identification using real spatial omics datasets. Hierarchical tissue structure identification under different model configurations using **(a)** the mouse spleen CODEX dataset (imaging-based) and **(b)** the human colorectal cancer (CRC) Visium HD dataset (sequencing-based). Each run was run 10 times with different random seeds. Boxplot elements are defined as: center line, median; box limits, upper and lower quartiles; whiskers, 1.5× interquartile range. Performances are quantified by Adjusted Mutual Information (AMI) and Macro-F1 scores. Each point corresponds to the performance of one run. This sensitivity analysis includes five hyperparameters: (1) the DropNode rate (fraction of nodes whose features are masked during consistency regularization training), (2) λ_consis (weight of the consistency regularization term), (3) λ_balance (weight of the balance regularization term), (4) the number of neighbors *K* in the KNN graph (cell-cell proximity graph) construction, and (5) the number of perturbations used for

generating perturbed cell-cell proximity graphs. Source data are provided as a Source Data file.

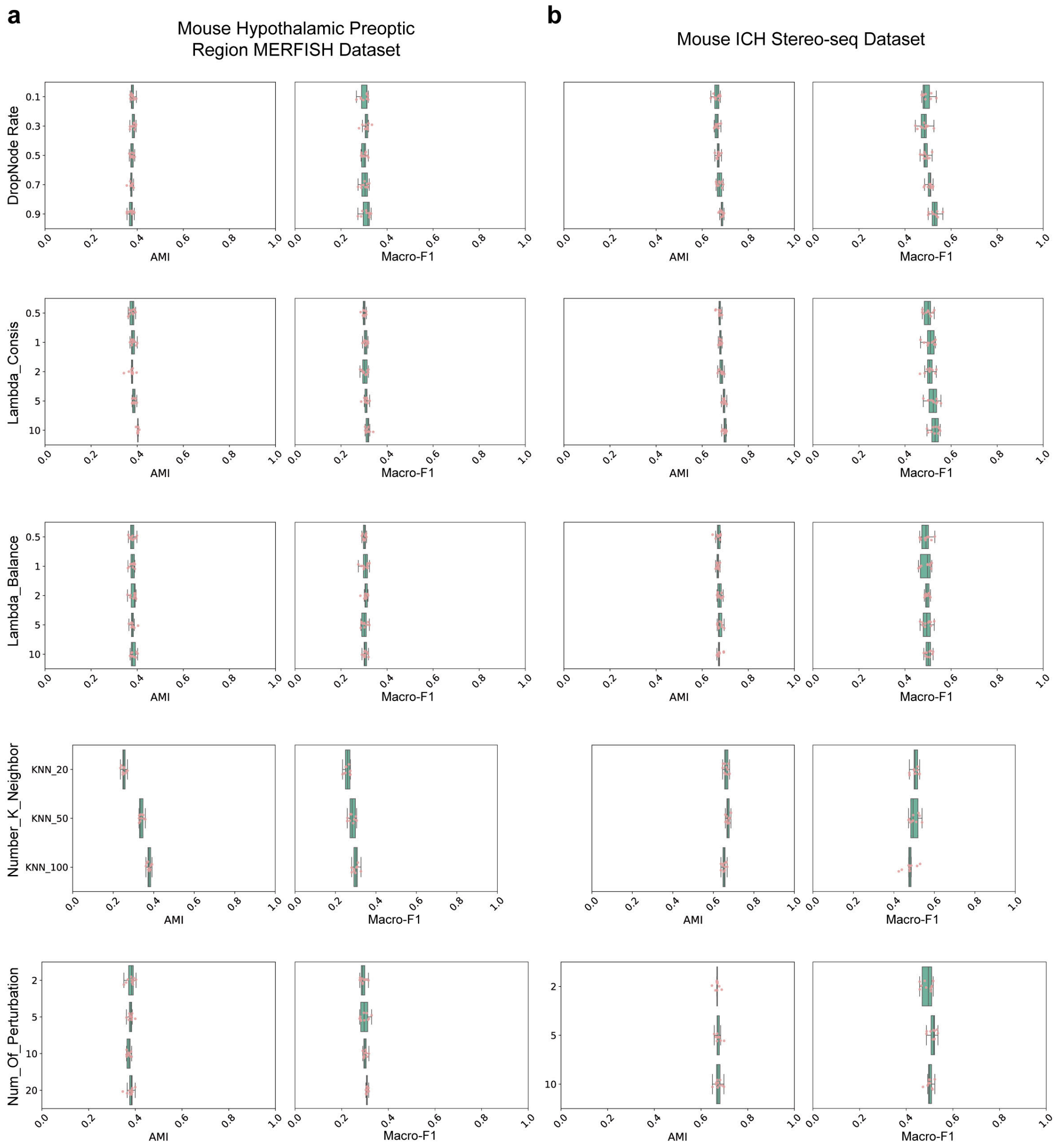

Supplementary Figure 18. Sensitivity analysis of hyperparameters in HRCHY-CytoCommunity on fine-grained CN identification using real spatial omics datasets. Hierarchical tissue structure identification under different model configurations using **(a)** the mouse hypothalamic preoptic region MERFISH dataset (imaging-based) and **(b)** the mouse intracerebral hemorrhage (ICH) Stereo-seq dataset (sequencing-based). Each configuration was run 10 times with different random seeds. Boxplot elements are defined as: center line, median; box limits, upper and lower quartiles; whiskers, 1.5× interquartile range. Performances are quantified by Adjusted Mutual Information (AMI) and Macro-F1 scores. Each point corresponds to the performance of one run. This sensitivity analysis includes five hyperparameters: (1) the DropNode rate (fraction of nodes whose features are masked during consistency regularization training), (2) λ_consis (weight of the consistency regularization term), (3) λ_balance (weight of the balance regularization term), (4) the number of neighbors *K* in the KNN graph (cell-cell proximity graph) construction,

and (5) the number of perturbations used for generating perturbed cell-cell proximity graphs. Source data are provided as a Source Data file.

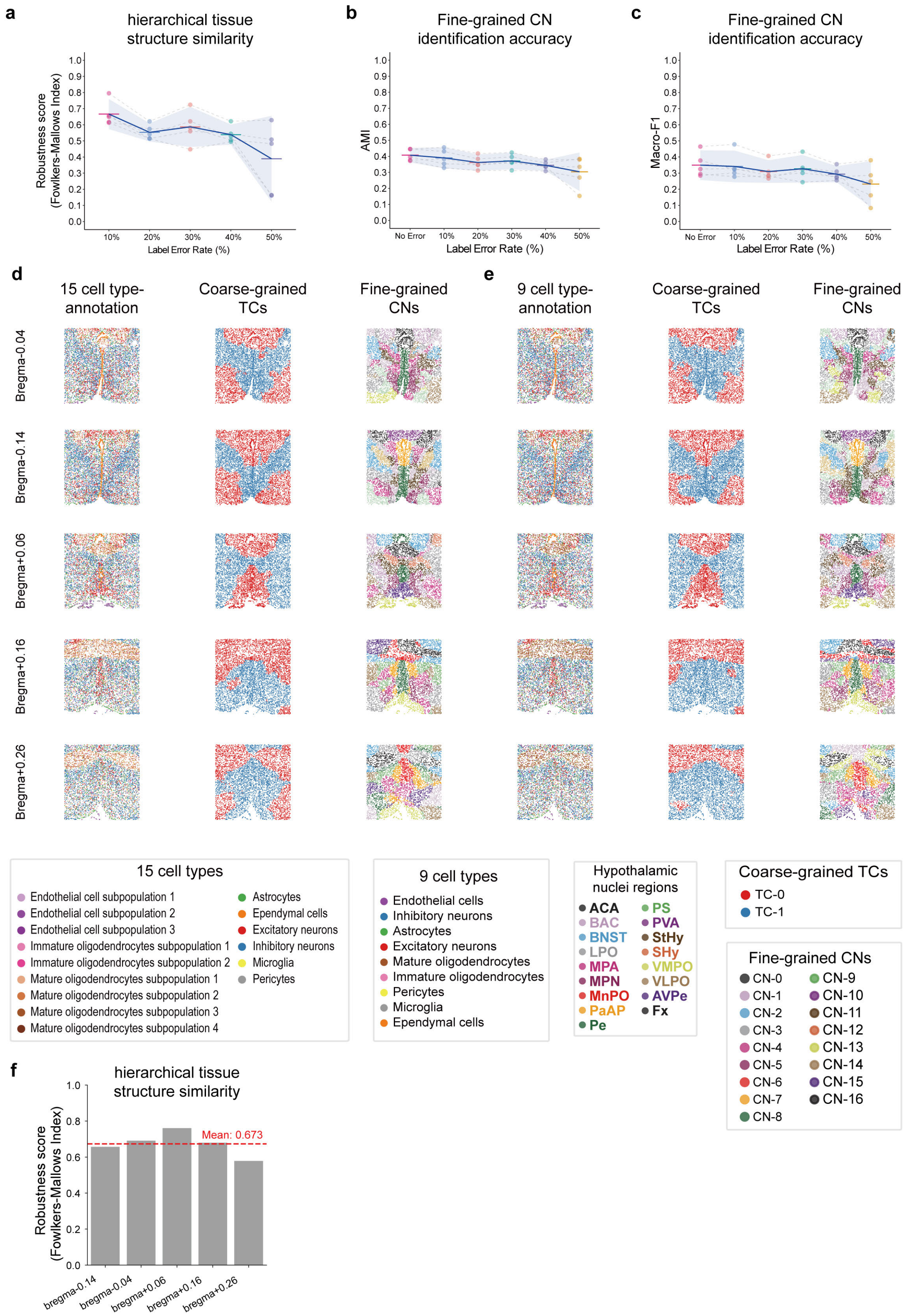

Supplementary Figure 19. Robustness assessment of HRCHY-CytoCommunity to label inaccuracies and annotation resolution variations using the mouse hypothalamic preoptic region MERFISH dataset. **(a)** Hierarchical tissue structure similarity under increasing cell-type label perturbation rates (10%-50%), quantified by Fowlkes-Mallows Index (FMI)-based robustness score. Higher values indicate

greater structural consistency with the unperturbed reference. **(b-c)** Fine-grained CN identification accuracy under label perturbations, measured by Adjusted Mutual Information (AMI) **(b)** and Macro-F1 scores **(c)**. Each point represents performance on one sample, with horizontal bars represent the mean performance across n = 5 samples. Performances (points) on the same sample are connected by grey dashed lines. Mean values under each label error rate are connected by blue solid lines. Shaded areas denote standard deviation. **(d)** Single-cell spatial maps showing the distribution of 15 cell types (left), coarse-grained TCs (middle) and fine-grained CNs (right) identified by HRCHY-CytoCommunity based on this high-resolution annotation. **(e)** Single-cell spatial maps showing the distribution of 9 cell types (left), coarse-grained TCs (middle) and fine-grained CNs (right) identified by HRCHY-CytoCommunity based on this low-resolution annotation. **(f)** Hierarchical tissue structure similarity between different cell-type annotation resolutions, quantified by FMI-based robustness score. The red dashed line indicates the mean similarity (0.673) relative to the 15-cell-type annotation-based reference. Source data are provided as a Source Data file.

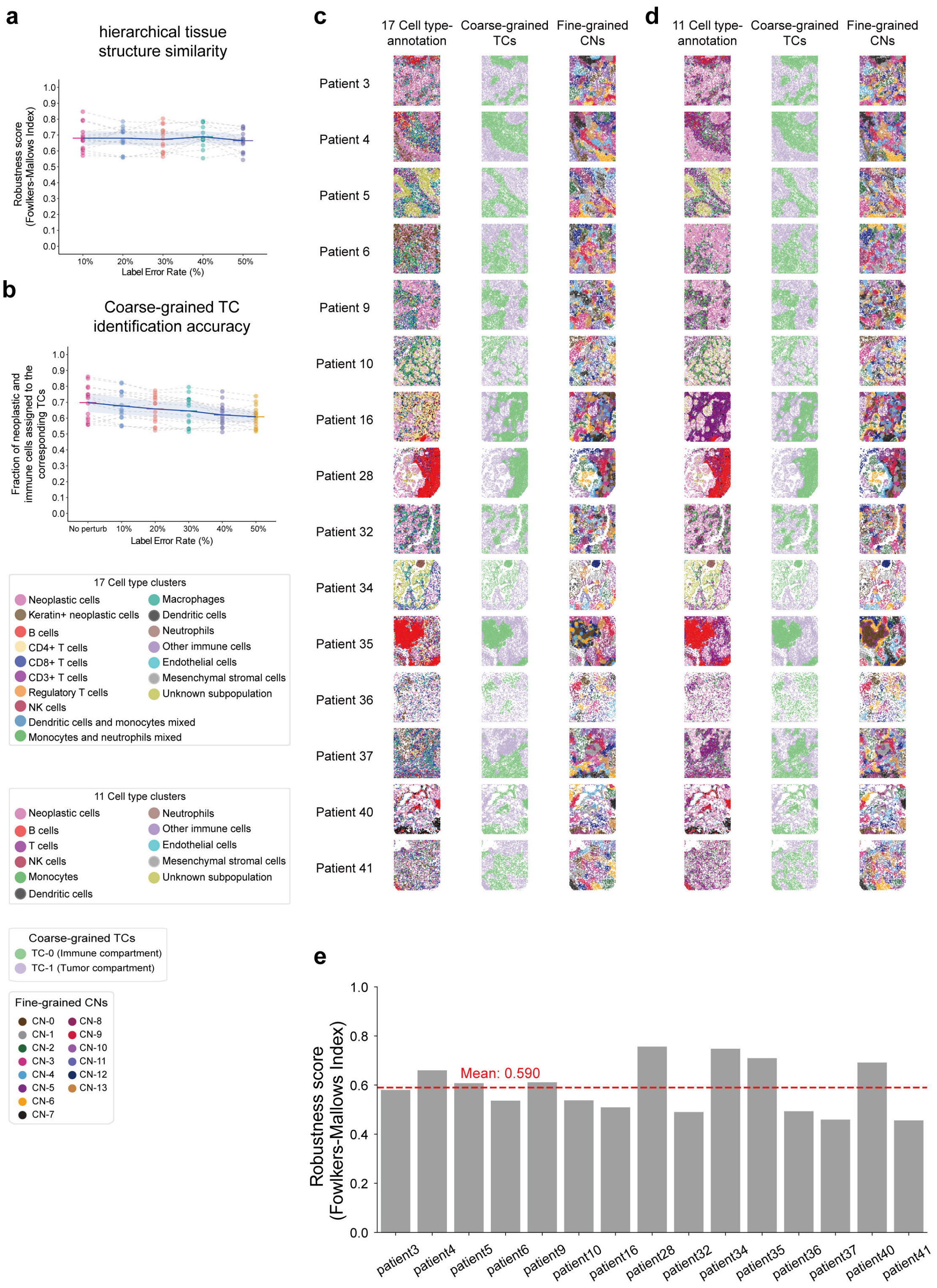

Supplementary Figure 20. Robustness assessment of HRCHY-CytoCommunity to label inaccuracies and annotation resolution variations using the human TNBC MIBI-TOF dataset. (a) Hierarchical tissue structure similarity under increasing cell-type label perturbation rates (10%-50%), quantified by Fowlkes-Mallows Index (FMI)-based robustness score. Higher values indicate greater structural consistency with the unperturbed reference. **(b)** Coarse-grained TC identification accuracy under label perturbations, measured by proportion of

neoplastic and immune cells correctly assigned to their corresponding TCs. Each point represents performance on one compartmentalized tumor sample, with horizontal bars represent the mean performance across n = 15 samples. Performances (points) on the same sample are connected by grey dashed lines. Mean values under each label error rate are connected by blue solid lines. Shaded areas denote standard deviation. **(c)** Single-cell spatial maps showing the distribution of 17 cell types (left), coarse-grained TCs (middle) and fine-grained CNs (right) identified by HRCHY-CytoCommunity based on this high-resolution annotation. **(d)** Single-cell spatial maps showing the distribution of 11 cell types (left), coarse-grained TCs (middle) and fine-grained CNs (right) identified by HRCHY-CytoCommunity based on this low-resolution annotation. **(e)** Hierarchical tissue structure similarity between different cell-type annotation resolutions, quantified by FMI-based robustness score. The red dashed line indicates the mean similarity (0.590) relative to the 17-cell-type annotation-based reference. Source data are provided as a Source Data file.

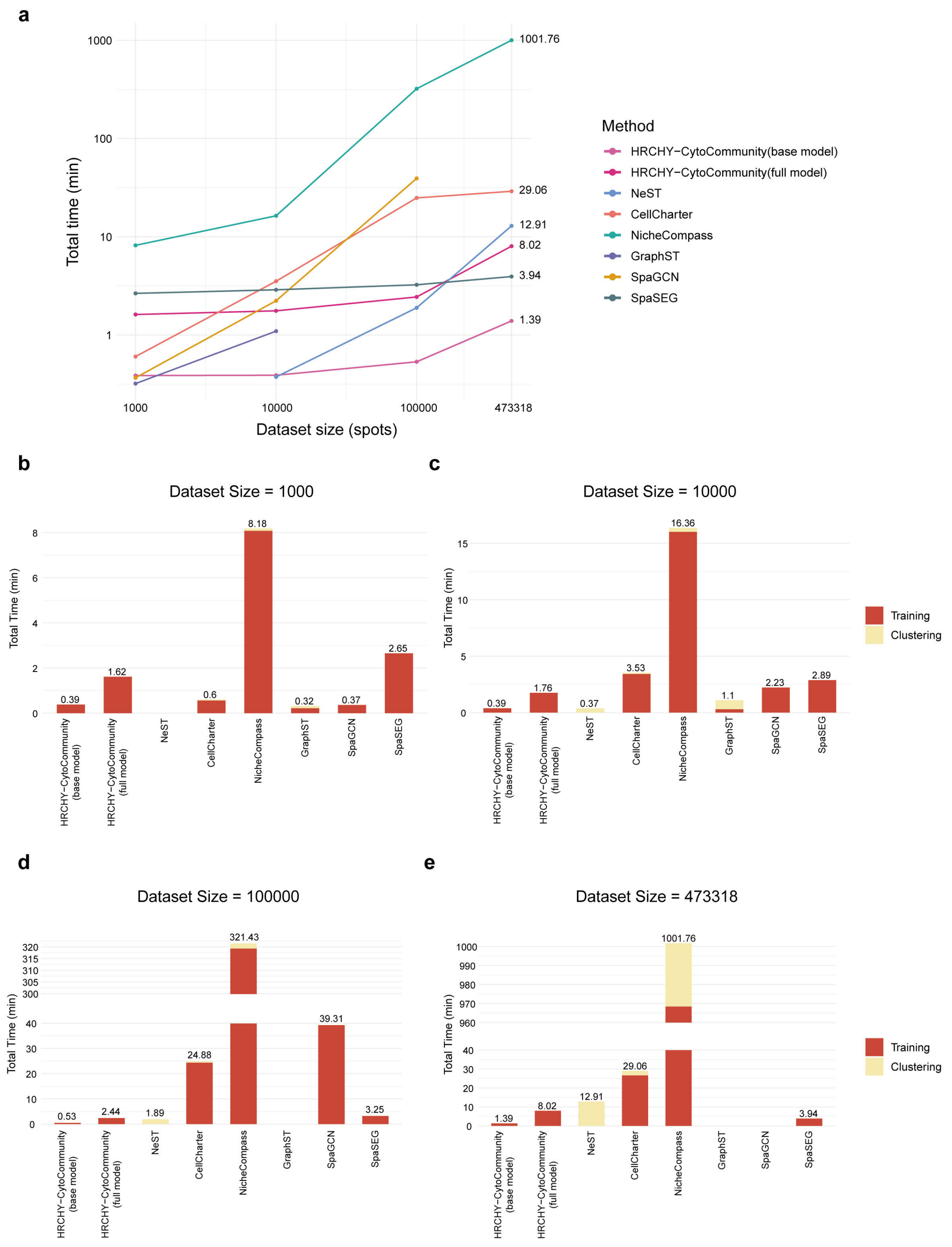

Supplementary Figure 21. Runtime benchmarking using Visium HD datasets. (a) Total runtime of eight methods, including the base and full models of HRCHY-CytoCommunity, NeST, CellCharter, NicheCompass, GraphST, SpaGCN, and SpaSEG, evaluated on datasets ranging from 1k to 473k spots. **(b-e)** Breakdown of runtime into training (red) and clustering (yellow) stages for each method, shown for datasets containing 1k **(b)**, 10k **(c)**, 100k **(d)**, and 473k **(e)** spots. Source data are provided as a Source Data file.

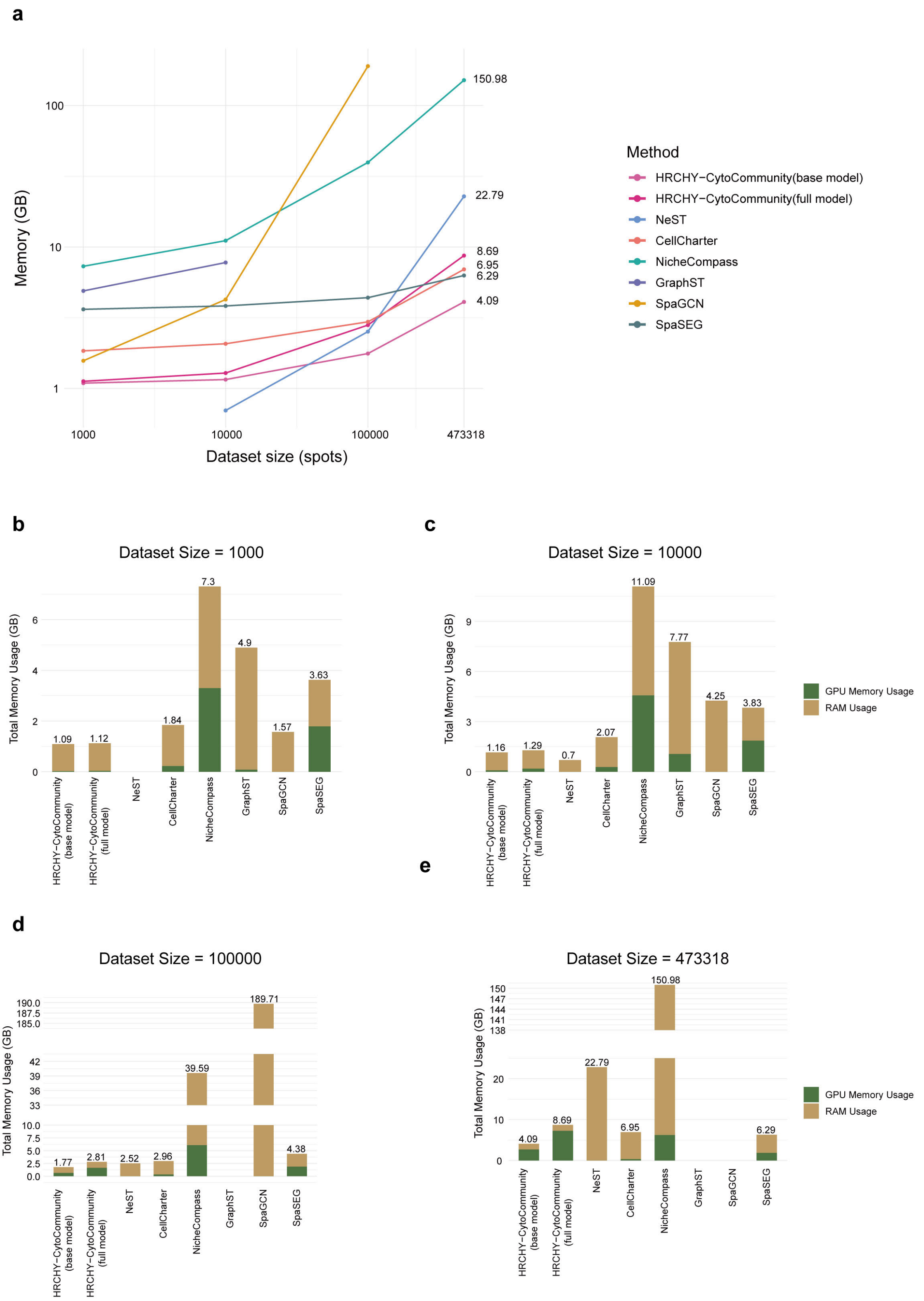

Supplementary Figure 22. Memory usage benchmarking using Visium HD datasets. (a) Total memory consumption of eight methods, including both base and full models of HRCHY-CytoCommunity, NeST, CellCharter, NicheCompass, GraphST, SpaGCN, and SpaSEG, evaluated on datasets ranging from 1k to 473k spots. **(b-e)** Breakdown of total memory into GPU memory (green) and RAM (brown)

usage for each method, shown for datasets containing 1k **(b)**, 10k **(c)**, 100k **(d)**, and 473k **(e)** spots. Source data are provided as a Source Data file.

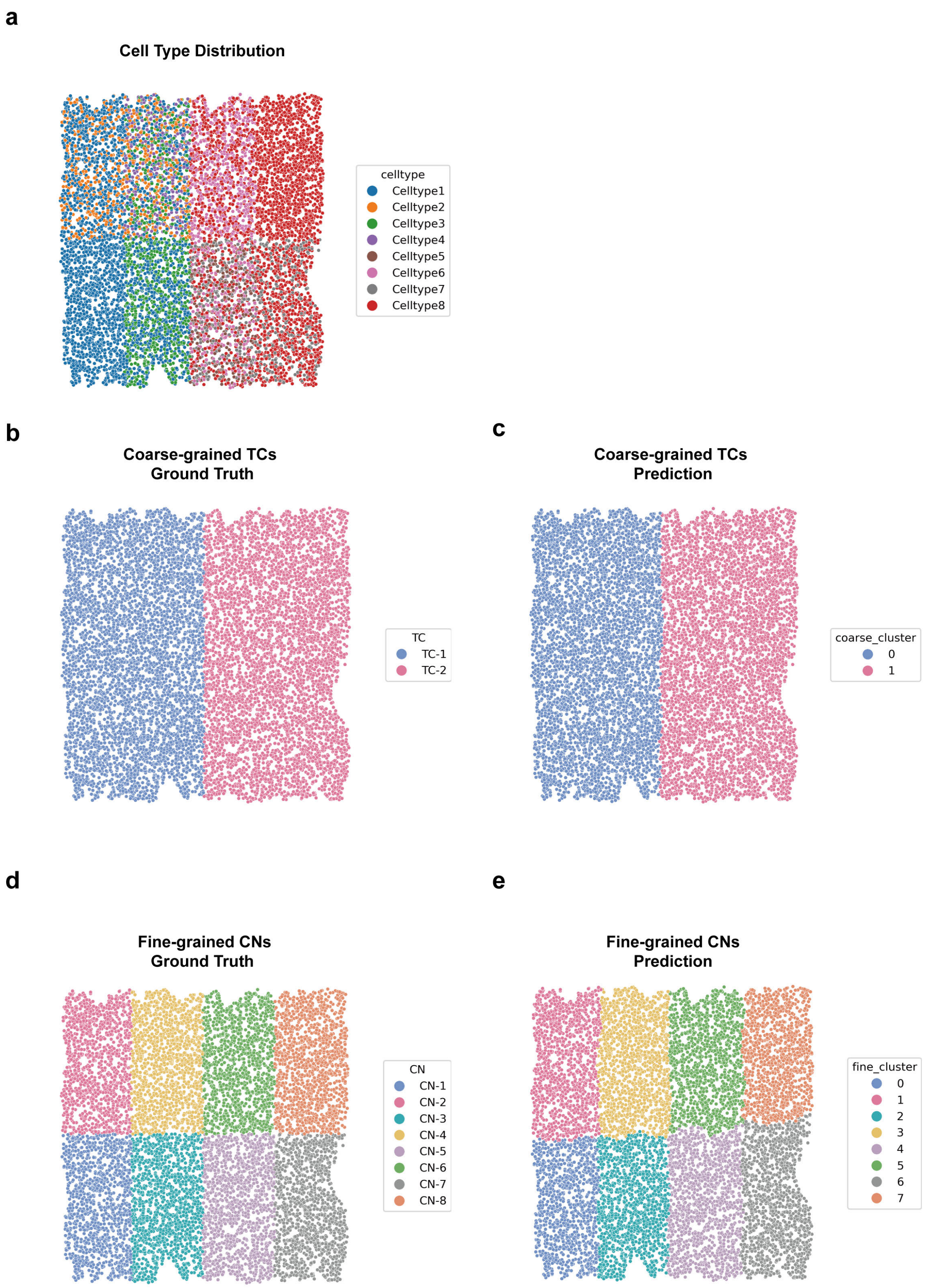

Supplementary Figure 23. Performance evaluation of HRCHY-CytoCommunity using the simulated dataset. (a) Spatial distribution of eight cell types in the simulated dataset. **(b)** Ground truth of the coarse-grained TCs. **(c)** Coarse-grained TCs identified by HRCHY-CytoCommunity (AMI = 0.956, Macro-F1 score = 0.994). **(d)** Ground truth of the fine-grained CNs**. (e)** Fine-grained CNs identified by HRCHY-CytoCommunity (AMI = 0.914, Macro-F1 score = 0.959).

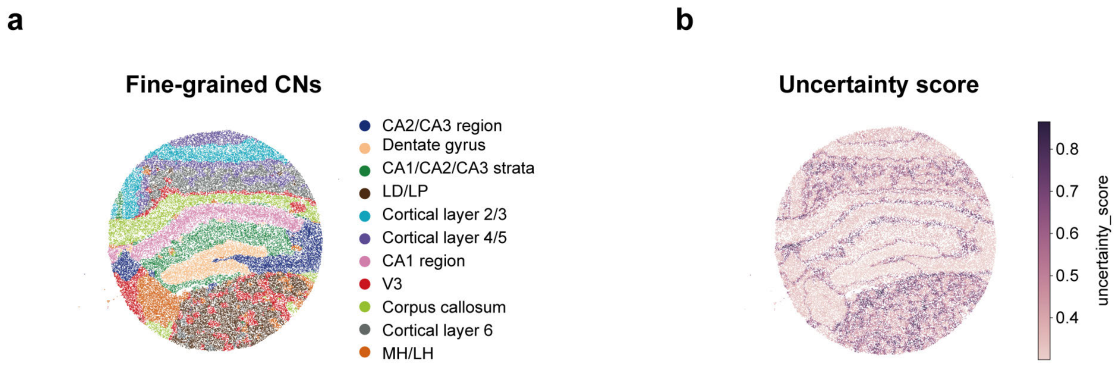

Supplementary Figure 24. Ambiguous structures in the mouse Slide-seq V2 dataset. (a) Fine-grained CNs identified by HRCHY-CytoCommunity. **(b)** Uncertainty score distribution, with values near 0 indicating high confidence in tissue structure assignment and values near 1 reflecting high ambiguity.

Supplementary Tables

Supplementary Table 1. Hyperparameter settings of HRCHY-CytoCommunity used in all datasets.

<table>
<tr><th>Dataset</th><th>#TC</th><th>#CN</th><th>#K</th><th>#GNN layers</th><th>#MLP layers</th><th>#Epochs</th><th>λ_{consis}</th><th>$\lambda_{balance}$</th><th>#Perturbations</th><th>Drop Node rate</th><th>Learning rate</th></tr>
<tr><td>Human CRC Visium HD dataset</td><td>2</td><td>10</td><td>20</td><td rowspan="8">2</td><td rowspan="8">2</td><td rowspan="8">1500</td><td rowspan="8">1</td><td rowspan="8">1</td><td rowspan="8">5</td><td rowspan="8">0.5</td><td rowspan="8">0.0001</td></tr>
<tr><td>Human Breast Cancer Visium dataset</td><td>2</td><td>9</td><td>20</td></tr>
<tr><td>Mouse ICH Stereo-seq dataset</td><td>3</td><td>20</td><td>50</td></tr>
<tr><td>Mouse spleen CODEX dataset</td><td>2</td><td>7</td><td>50</td></tr>
<tr><td>Mouse hippocampus Slide-seq V2 dataset</td><td>3</td><td>11</td><td>50</td></tr>
<tr><td>Mouse hypothalamic preoptic region MERFISH dataset</td><td>2</td><td>12</td><td>100</td></tr>
<tr><td>Human TNBC MIBI-TOF dataset</td><td>2</td><td>14</td><td>50</td></tr>
<tr><td>Human Breast Cancer IMC dataset</td><td>2</td><td>7</td><td>20</td></tr>
</table>

This table details the configuration of the HRCHY-CytoCommunity model for each dataset, including the number of tissue compartments (TCs) and cellular neighborhoods (CNs), the value of *K* in the *K*-nearest neighbor graph, the number of GNN and MLP layers, total training epochs, regularization weights (α, λ_*consis*, and λ_*balance*), the number of perturbations on the cell-cell proximity graph, DropNode rate, and learning rate.

Supplementary Table 2. Hyperparameter settings of NeST used in all datasets.

Dataset	ϵ	Min samples	Hotspot min size	Jaccard threshold	Min size	Cutoff	Min genes	Resolution
Mouse spleen CODEX dataset	400	100	50	0.1	50	0.1	2	1
Mouse hypothalamic preoptic region MERFISH dataset	300	2	2	0.1	10	0.1	2	1
Mouse ICH Stereo-seq dataset	300	5	50	0.1	50	0.1	5	1
Human CRC Visium HD dataset	300	4	15	0.3	10	0.1	3	1
Human Breast Cancer Visium dataset	300	4	15	0.6	10	0.3	15	1
Mouse hippocampus Slide-seq V2 dataset	75	5	50	0.35	30	0.5	8	1
Human TNBC TIBI-TOF dataset	100	4	5	0.3	10	0.3	2	1

Supplementary Table 3. Cell-type composition profiles for CNs in simulation data.

	Celltype1	Celltype2	Celltype3	Celltype4	Celltype5	Celltype6	Celltype7	Celltype8
CN-1	1	0	0	0	0	0	0	0
CN-2	0.7	0.3	0	0	0	0	0	0
CN-3	0.5	0	0.5	0	0	0	0	0
CN-4	0.25	0.25	0.25	0.25	0	0	0	0
CN-5	0	0	0	0	0.25	0.25	0.25	0.25
CN-6	0	0	0	0	0	0.5	0	0.5
CN-7	0	0	0	0	0	0	0.3	0.7
CN-8	0	0	0	0	0	0	0	1

Supplementary References

1. Chen, R., Peng, B., Zhu, P. & Wang, Y. Editorial: Modulation of neuronal excitability by non-neuronal cells in physiological and pathophysiological conditions. *Front. Cell. Neurosci.* **17**, (2023).
2. Stevenson, R., Samokhina, E., Rossetti, I., Morley, J. W. & Buskila, Y. Neuromodulation of Glial Function During Neurodegeneration. *Front Cell Neurosci* **14**, 278 (2020).
3. Shi, J.-M. *et al.* Identification and functional comparison of primary astrocytes and microglia. *J Neurosci Methods* **383**, 109731 (2023).
