# Supplementary material for: HRCHY-CytoCommunity identifies hierarchical tissue organization in cell-type spatial maps
Source: Nat Commun. 2026 Feb 28;17:3312. doi: 10.1038/s41467-026-70069-z (PMC13065825; doi:10.1038/s41467-026-70069-z)
Supplement: Supplementary file 2 — Description of Additional Supplementary Files [file 41467_2026_70069_MOESM2_ESM.pdf]

Description of Additional Supplementary Files:

Supplementary Data 1: Detailed information of datasets used in this study
