# Supplementary material for: HRCHY-CytoCommunity identifies hierarchical tissue organization in cell-type spatial maps
Source: Nat Commun. 2026 Feb 28;17:3312. doi: 10.1038/s41467-026-70069-z (PMC13065825; doi:10.1038/s41467-026-70069-z)
Supplement: Supplementary file 4 — Reporting Summary [file 41467_2026_70069_MOESM4_ESM.pdf]

nature portfolio

Corresponding author(s): Lin Gao, Yuxuan Hu, Jiadong Lin

Last updated by author(s): Jan 7, 2026

Reporting Summary

Nature Portfolio wishes to improve the reproducibility of the work that we publish. This form provides structure for consistency and transparency in reporting. For further information on Nature Portfolio policies, see our Editorial Policies and the Editorial Policy Checklist.

Statistics

For all statistical analyses, confirm that the following items are present in the figure legend, table legend, main text, or Methods section.

n/a	Confirmed	
☐	☒	The exact sample size (*n*) for each experimental group/condition, given as a discrete number and unit of measurement
☐	☒	A statement on whether measurements were taken from distinct samples or whether the same sample was measured repeatedly
☐	☒	The statistical test(s) used AND whether they are one- or two-sided<br>*Only common tests should be described solely by name; describe more complex techniques in the Methods section.*
☐	☒	A description of all covariates tested
☐	☒	A description of any assumptions or corrections, such as tests of normality and adjustment for multiple comparisons
☐	☒	A full description of the statistical parameters including central tendency (e.g. means) or other basic estimates (e.g. regression coefficient) AND variation (e.g. standard deviation) or associated estimates of uncertainty (e.g. confidence intervals)
☐	☒	For null hypothesis testing, the test statistic (e.g. *F*, *t*, *r*) with confidence intervals, effect sizes, degrees of freedom and *P* value noted<br>*Give P values as exact values whenever suitable.*
☒	☐	For Bayesian analysis, information on the choice of priors and Markov chain Monte Carlo settings
☒	☐	For hierarchical and complex designs, identification of the appropriate level for tests and full reporting of outcomes
☒	☐	Estimates of effect sizes (e.g. Cohen's *d*, Pearson's *r*), indicating how they were calculated

Our web collection on statistics for biologists contains articles on many of the points above.

Software and code

Policy information about availability of computer code

Data collection	No software was used to collect data.
Data analysis	The method HRCHY-CytoCommunity proposed in this paper has been deposited at GitHub (https://github.com/huBioinfo/HRCHY-CytoCommunity). The code base for running NeST was downloaded from https://github.com/bwalker1/NeST. The code base for running SpaSEG was downloaded from https://github.com/y-bai/SpaSEG.Other compared methods are GraphST (v.1.1.1), NicheCompass (v.0.3.0), CellCharter (v.0.3.5), SpaGCN (v.1.2.7), and Giotto Suite (v4.2.1).The data analysis tools used include the Python package "scikit-learn (v1.1.2)" and the R package "survival (v3.6-4)" and "spacexr (v2.2.0)".

For manuscripts utilizing custom algorithms or software that are central to the research but not yet described in published literature, software must be made available to editors and reviewers. We strongly encourage code deposition in a community repository (e.g. GitHub). See the Nature Portfolio guidelines for submitting code & software for further information.

Data

Policy information about availability of data

All manuscripts must include a data availability statement. This statement should provide the following information, where applicable:

- Accession codes, unique identifiers, or web links for publicly available datasets
- A description of any restrictions on data availability
- For clinical datasets or third party data, please ensure that the statement adheres to our policy

All data used in this study are publicly available. This study used the following eight publicly available spatial omics datasets, including a mouse spleen CODEX

dataset (https://data.mendeley.com/datasets/zjnpwh8m5b/1), a mouse hypothalamic preoptic region MERFISH dataset (https://datadryad.org/stash/dataset/doi:10.5061/dryad.8t8s248), a human triple-negative breast cancer MIBI-TOF dataset (https://mibi-share.ionpath.com), a human breast cancer IMC dataset (https://zenodo.org/record/3518284#.Y2UQ0-xBybg), a human CRC Visium HD dataset (https://www.10xgenomics.com/platforms/visium/product-family/dataset-human-crc), a human breast cancer Visium V1 dataset (https://www.10xgenomics.com/datasets/human-breast-cancer-block-a-section-1-1-standard-1-1-0), a mouse hippocampus Slide-seq V2 dataset (https://singlecell.broadinstitute.org/single_cell/study/SCP815/highly-sensitive-spatial-transcriptomics-at-near-cellular-resolution-with-slide-seqv2), and a mouse ICH Stereo-seq dataset (https://db.cngb.org/stomics/stmich/). Two single-cell transcriptomic datasets were used as references for cell-type deconvolution in the spot-based spatial transcriptomics data, including a human breast cancer scRNA-seq dataset (GEO: GSE176078; https://www.ncbi.nlm.nih.gov/geo/query/acc.cgi?acc=GSE176078), and a mouse hippocampus scRNA-seq dataset (https://singlecell.broadinstitute.org/single_cell/study/SCP948/robust-decomposition-of-cell-type-mixtures-in-spatial-transcriptomics#study-download). No new data were generated in this study.

Research involving human participants, their data, or biological material

Policy information about studies with human participants or human data. See also policy information about sex, gender (identity/presentation), and sexual orientation and race, ethnicity and racism.

Reporting on sex and gender	N/A
Reporting on race, ethnicity, or other socially relevant groupings	N/A
Population characteristics	N/A
Recruitment	N/A
Ethics oversight	N/A

Note that full information on the approval of the study protocol must also be provided in the manuscript.

Field-specific reporting

Please select the one below that is the best fit for your research. If you are not sure, read the appropriate sections before making your selection.

[x] Life sciences   [ ] Behavioural & social sciences   [ ] Ecological, evolutionary & environmental sciences

For a reference copy of the document with all sections, see nature.com/documents/nr-reporting-summary-flat.pdf

Life sciences study design

All studies must disclose on these points even when the disclosure is negative.

Sample size	The mouse spleen CODEX dataset includes three samples with ground-truth annotations. The human colorectal cancer Visium HD dataset includes three samples with ground-truth annotations. The mouse hypothalamic preoptic region MERFISH dataset includes five samples with ground-truth annotations. The mouse intracerebral hemorrhage Stereo-seq dataset includes nine samples with ground-truth annotations. The mouse hippocampus Slide-seq V2 dataset includes one sample. The human breast cancer Visium V1 dataset includes one sample. The human triple-negative breast cancer MIBI-TOF dataset includes 15 compartmentalized tumor samples, in which immune cells and tumor cells are spatially separated and can be used for quantitative assessment. The human breast cancer IMC dataset includes 263 breast cancer patients, with 190 surviving and 73 deceased, which can verify the biological significance and clinical relevance of the hierarchical tissue structures identified by HRCHY-CytoCommunity through the survival data of patients.
Data exclusions	No data were excluded from the analyses.
Replication	We repeatedly conducted the analysis of each dataset and all attempts at replication were successful.
Randomization	All the samples were allocated into experimental groups.
Blinding	Blinding is not applicable to this study because our main finding is the development of a computational tool named HRCHY-CytoCommunity, and we used publicly available datasets for all data analyses.

Reporting for specific materials, systems and methods

We require information from authors about some types of materials, experimental systems and methods used in many studies. Here, indicate whether each material, system or method listed is relevant to your study. If you are not sure if a list item applies to your research, read the appropriate section before selecting a response.

Materials & experimental systems

n/a	Involved in the study
☒	☐ Antibodies
☒	☐ Eukaryotic cell lines
☒	☐ Palaeontology and archaeology
☒	☐ Animals and other organisms
☒	☐ Clinical data
☒	☐ Dual use research of concern
☒	☐ Plants

Methods

n/a	Involved in the study
☒	☐ ChIP-seq
☒	☐ Flow cytometry
☒	☐ MRI-based neuroimaging

Plants

Seed stocks	N/A
Novel plant genotypes	N/A
Authentication	N/A
